# *Staphylococcus epidermidis* bacteriocin A37 kills natural competitors with a unique mechanism of action

**DOI:** 10.1093/ismejo/wrae044

**Published:** 2024-03-12

**Authors:** Jan-Samuel Puls, Benjamin Winnerling, Jeffrey J Power, Annika M Krüger, Dominik Brajtenbach, Matthew Johnson, Kevser Bilici, Laura Camus, Thomas Fließwasser, Tanja Schneider, Hans-Georg Sahl, Debnath Ghosal, Ulrich Kubitscheck, Simon Heilbronner, Fabian Grein

**Affiliations:** Institute for Pharmaceutical Microbiology, University Hospital Bonn, University of Bonn, 53115 Bonn, Germany; Institute for Pharmaceutical Microbiology, University Hospital Bonn, University of Bonn, 53115 Bonn, Germany; German Center for Infection Research (DZIF), Partner Site Bonn-Cologne, 53115 Bonn, Germany; Interfaculty Institute of Microbiology and Infection Medicine, Department of Infection Biology, University of Tübingen, 72076 Tübingen, Germany; Clausius Institute of Physical and Theoretical Chemistry, University of Bonn, 53115 Bonn, Germany; Clausius Institute of Physical and Theoretical Chemistry, University of Bonn, 53115 Bonn, Germany; Department of Biochemistry and Pharmacology, Bio21 Molecular Science and Biotechnology Institute, The University of Melbourne, Melbourne, VIC 3010, Australia; ARC Centre for Cryo-electron Microscopy of Membrane Proteins, Bio21 Molecular Science and Biotechnology Institute, University of Melbourne, Parkville, VIC 3010, Australia; Interfaculty Institute of Microbiology and Infection Medicine, Department of Infection Biology, University of Tübingen, 72076 Tübingen, Germany; Interfaculty Institute of Microbiology and Infection Medicine, Department of Infection Biology, University of Tübingen, 72076 Tübingen, Germany; Institute for Pharmaceutical Microbiology, University Hospital Bonn, University of Bonn, 53115 Bonn, Germany; German Center for Infection Research (DZIF), Partner Site Bonn-Cologne, 53115 Bonn, Germany; Institute for Pharmaceutical Microbiology, University Hospital Bonn, University of Bonn, 53115 Bonn, Germany; German Center for Infection Research (DZIF), Partner Site Bonn-Cologne, 53115 Bonn, Germany; Institute for Pharmaceutical Microbiology, University Hospital Bonn, University of Bonn, 53115 Bonn, Germany; Department of Biochemistry and Pharmacology, Bio21 Molecular Science and Biotechnology Institute, The University of Melbourne, Melbourne, VIC 3010, Australia; ARC Centre for Cryo-electron Microscopy of Membrane Proteins, Bio21 Molecular Science and Biotechnology Institute, University of Melbourne, Parkville, VIC 3010, Australia; Clausius Institute of Physical and Theoretical Chemistry, University of Bonn, 53115 Bonn, Germany; Interfaculty Institute of Microbiology and Infection Medicine, Department of Infection Biology, University of Tübingen, 72076 Tübingen, Germany; German Centre for Infection Research (DZIF), Partner Site Tübingen, 72076 Tübingen, Germany; Present address: Faculty of Biology, Microbiology, Ludwig-Maximilians-University of Munich, 82152 München, Germany; Institute for Pharmaceutical Microbiology, University Hospital Bonn, University of Bonn, 53115 Bonn, Germany; German Center for Infection Research (DZIF), Partner Site Bonn-Cologne, 53115 Bonn, Germany

**Keywords:** bacteriocins, lantibiotics, epilancins, mechanism of action, corynebacteria, staphylococci

## Abstract

Many bacteria produce antimicrobial compounds such as lantibiotics to gain advantage in the competitive natural environments of microbiomes. Epilancins constitute an until now underexplored family of lantibiotics with an unknown ecological role and unresolved mode of action. We discovered production of an epilancin in the nasal isolate *Staphylococcus epidermidis* A37. Using bioinformatic tools, we found that epilancins are frequently encoded within staphylococcal genomes, highlighting their ecological relevance. We demonstrate that production of epilancin A37 contributes to *Staphylococcus epidermidis* competition specifically against natural corynebacterial competitors. Combining microbiological approaches with quantitative *in vivo* and *in vitro* fluorescence microscopy and cryo-electron tomography, we show that A37 enters the corynebacterial cytoplasm through a partially transmembrane-potential-driven uptake without impairing the cell membrane function. Upon intracellular aggregation, A37 induces the formation of intracellular membrane vesicles, which are heavily loaded with the compound and are essential for the antibacterial activity of the epilancin. Our work sheds light on the ecological role of epilancins for staphylococci mediated by a mode of action previously unknown for lantibiotics.

## Introduction

In the hostile environments of the human skin and nose, bacteria must compete for limited nutrients and space [1, 2]. To proliferate within these highly competitive environments, many microorganisms engage in a “bacterial warfare” by producing a variety of compounds that provide a competitive advantage. Part of the antibacterial arsenal are bacteriocins, a large and broadly defined group of primarily small molecule peptides that kill or inhibit the growth of other bacteria [3].

Lantibiotics constitute a large subgroup of bacteriocins with well over 80 described compounds [4]. They are posttranslationally modified peptides characterized by lanthionine groups as the defining structural motif. The described mechanisms of action are either based on peptidoglycan synthesis inhibition or membrane disruption via different modes of interaction. Still, the precise mechanism of antibacterial activity and the ecological function is unknown for many lantibiotics [5–7].

The first discovered and best-researched lantibiotic is nisin, which uses the ultimate peptidoglycan precursor molecule Lipid II as a high-affinity membrane target to facilitate pore formation [8–13]. Because of its early discovery, relevance as a food preservative, and potential for medical use, the structural–functional relationship of nisin has been extensively researched. Nisin features five thioether ring systems, of which the N-terminal Rings A–B are essential for binding the cell wall precursor Lipid II [9, 14]. The three Rings C–E were shown to interact with and penetrate the cytoplasmic membrane and facilitate the formation of nisin:Lipid II complexes, causing pore formation, complex aggregation, membrane disintegration, and ultimately cell death [10–15].

Epilancins are a subgroup of lantibiotics produced by staphylococci with close structural similarity to nisin [5]. The C-terminal and middle regions display nearly identical ring system configurations, albeit with some alterations in their amino acid sequence. However, the N-terminal two-ring motif in nisin responsible for Lipid II binding is absent in epilancins [16, 17]. Instead, a linear amino acid sequence of similar length is present and accordingly, epilancins show no specific interaction with Lipid II [5, 8]. Given the striking similarity of the C-terminal region to that of nisin, membrane damaging mechanisms have been proposed, but inconsistent data did not allow for a general mechanism of action (MoA) hypothesis [8, 18–21].

The unclear ecological role of epilancins led to speculations about a potential microbiome-shaping function given the high abundance of staphylococci in the human microbiota [3, 22]. Staphylococci and corynebacteria are key members of the human nose and skin microbiota. Members of both genera are embedded in a complex interaction network governing microbiome composition [2, 23–25]. Here, they naturally compete for nutrients, space, and scarce resources. Corynebacteria evolved various strategies to inhibit expansion of staphylococci, whereas bacteriocins appear to be the main weapon in the arsenal of staphylococci [2, 3, 26–29]. Establishment of either genus within an ecological niche has been shown to modulate the pathogenic potential of relevant opportunistic species such as *Staphylococcus aureus, Streptococcus pneumoniae, Moraxella catarrhalis*, and others. This modulation can range from growth inhibition to influencing virulence factor expression or direct growth promotion [2, 24]. Additionally, both staphylococci and corynebacteria have considerable potential to cause severe to live threatening infections under certain conditions [22, 30–32]. This illustrates the complex balancing between beneficial and detrimental impact on microbiome and host health. Thus, unraveling key factors influencing the relationship between staphylococci and corynebacteria is essential to a comprehensive understanding of human microbiome ecology.

## Materials and methods

### Strains

All strains used in this work are listed in Table S1.

### Cultivation of bacteria

If not stated otherwise, Müller-Hinton Broth (Oxoid, UK) was used. For cultivation, overnight precultures were diluted 1:100 in identical medium. If not stated otherwise, cultures were grown at 37°C under constant shaking until they reached the desired optical density OD_600_ for the respective experiments.

### Isolation and purification of epilancin A37

Detailed description of isolation and purification can be found in the supplementary information.

### Serial passaging of *Corynebacterium glutamicum*

To facilitate the development of A37-resistant variants, an A37-sensitive strain of *C. glutamicum* was serially passaged over 21 passages. Each passage was performed following the procedure outlined under “Determination of Minimal Inhibitory Concentrations (MICs).” After Passage 1, for each subsequent passage, bacteria from the well with the highest concentration of A37 exhibiting growth were used as inoculum and the concentration range of A37 was increased depending on the MIC determined for the previous passage.

### Plate inhibition assays

Overnight cultures of corynebacteria were grown in 10-ml brain–heart infusion broth supplemented with 0.2% Tween 80 for 48 h at 37°C under constant shaking (160 rpm). *Staphylococcus epidermidis* was grown in 10-ml tryptic soy broth for 24 h at 37°C under constant shaking (160 rpm). All strains were washed once by resuspension in phosphate-buffered saline (PBS) and subsequent centrifugation (2 min, 13 000 rpm). Strains being tested for sensitivity (lawn strains) were adjusted to an optical density at 600 nm (OD_600_) of 0.05 and plated on BM medium [33] agar (1.5% European Agar) using a cotton swab. After drying for 5 min, 5 μl of the OD = 1 producer strain (*S. epidermidis*) culture was spotted on top of the lawn strain and dried for another 30 min. Areas of inhibitions surrounding the producer as well as the spot area formed by the producer were measured with ImageJ after 48-h growth at 37°C.

### Determination of MICs

MICs were determined by the serial broth dilution method in BD Difco Mueller Hinton broth (BD, USA) on 96-well plates according to CLSI standards. Briefly, bacteria at ~5 × 10^5^ cells per well were treated with increasing concentrations of A37 and incubated at appropriate temperatures. MICs were determined as the concentration of the first well that did not show any bacterial growth after 24 h of incubation.

### Competition *S. epidermidis*:*C. glutamicum*

Cultures of *S. epidermidis* and *C. glutamicum* were grown to mid-logarithmic growth phase and OD_600_ was adjusted to 0.05 with identical medium. These cell suspensions were mixed 99:1 (v/v, *C. glutamicum*:*S. epidermidis*) and incubated at 37°C and 120 rpm*.* After 3 h of co-incubation, cultures were mounted on 1% agarose slides and analyzed microscopically for relative abundance of each species, determined via phase contrast appearance.

### Growth curves *S. epidermidis* and *C. glutamicum*

Bacterial growth was quantified by measuring OD_600_. For growth curves of bacteria, 100 μL of culture were measured in sterile 96-well plates with u-shaped wells (Greiner Bio-One, AT) over time in 15-min intervals with regular shaking.

### Propidium iodide influx assays

Cultures were grown to OD_600_ = 0.25 and transferred into a flat bottom black polystyrene 96-well plate (Greiner Bio-One). Compound was added to Well 1 and diluted in a 1:2 dilution series and the plate was sealed and incubated at 37°C and 120 rpm. For the last 5 min of incubation, propidium iodide (PI; Thermo Fisher) was added in a final concentration of 10 μg/mL. At the end of incubation, the plate was centrifuged in a Heraeus Megafuge 40R (Heraeus, Germany) at 4500 rpm for 10 min. Cells were resuspended in sterile PBS at pH 7.4 and PI fluorescence was measured with a Tecan Spark 10 M (Tecan, CH) equipped with a monochromator with 530-nm excitation and 620-nm emission wavelengths.

### DiBAC_4_(3) influx assays

To quantify membrane depolarization using DiBAC_4_(3), cultures were grown to OD_600_ = 0.5, then compound was added. For the last 15 min of incubation, DiBAC_4_(3) (Biotium, USA) was added to a final concentration of 10 μM. Cultures were washed three times in medium (2 min, 13 000 rpm), mounted on 1% agarose slides and DiBAC_4_(3) fluorescence was analyzed microscopically.

### DiSC_3_(5) efflux assays

To measure DiSC_3_(5) efflux, cultures were grown to OD_600_ = 0.3. DiSC_3_(5) (Biomol, Germany) was added to a final concentration of 1 μM and the DMSO concentration was adjusted to 1% v/v. 200 μL of the culture was transferred into a Greiner Bio-One flat bottom black polystyrene 96-well plate. DiSC_3_(5) fluorescence was measured in a Tecan Spark 10 M equipped with a monochromator with 610-nm excitation and 660-nm emission wavelengths every minute until the signal remained stable for 5 min, indicating maximal uptake and auto-quenching of the dye. Compound was added and DiSC_3_(5) fluorescence was measured for at least 30 min.

### Whole-genome sequencing

The genomes of *S. epidermidis* A37 (NCBI accession number: SAMN36827138) and *Staphylococcus casei* K7 (formerly identified as *S. epidermidis* K7—NCBI accession number: SAMN36827139) were sequenced using long- and short-read sequencing. The resulting sequences can be found under NCBI BioProject PRJNA801128. Detailed description of whole-genome sequencing can be found in the supplementary information.

### Detection of epilancin gene clusters

We searched for epilancin gene clusters in all staphylococci assemblies from NCBI (downloaded February 2023). First, open reading frames with homology to the A37 cluster were identified using Prokka (v1.14.6, option –*proteins,* [34]). The *–proteins* option was used with an abbreviated GenBank file containing only the A37 cluster. Homologs were filtered for >70% coverage using blastp and > 50% completeness. Assemblies missing the gene encoding the precursor peptide were excluded. Finally, homologs were characterized based on protein similarity and integration site.

### Alignment and phylogenetic trees

The phylogenetic tree for the epilancin clusters (ECs) was calculated using the concatenated gene coding sequences for each assembly. *elaO* was omitted when aligning the concatenated sequences, as it is not present in all ECs. Additionally, EC14 was excluded from this analysis because of its markedly different composition compared with the other clusters. Concatenated sequences were aligned using the EMBL-EBI Clustal Omega tool [35], after which a phylogenetic tree was constructed using IQ-Tree [36]. Finally, the tree was visualized using Dendroscope [37].

Species trees were constructed using the full assembly sequences. Additional species (not containing an EC) were selected using Auto-MLST [38] and TYGS [39] to show the breadth and depth of the genetic diversity within the species. Final trees were calculated on TYGS using that list of species and visualized using Dendroscope.

Multi-sequence alignment of the precursor peptides was performed using Clustal Omega [35].

### Imaging and image data analysis

Detailed description of fluorescence microscopic methods, cryo-electron tomography, and image data analysis can be found in the supplementary information.

## Results

### Bacteriocin A37 is a member of the epilancin family of lantibiotics

**Figure 1:**
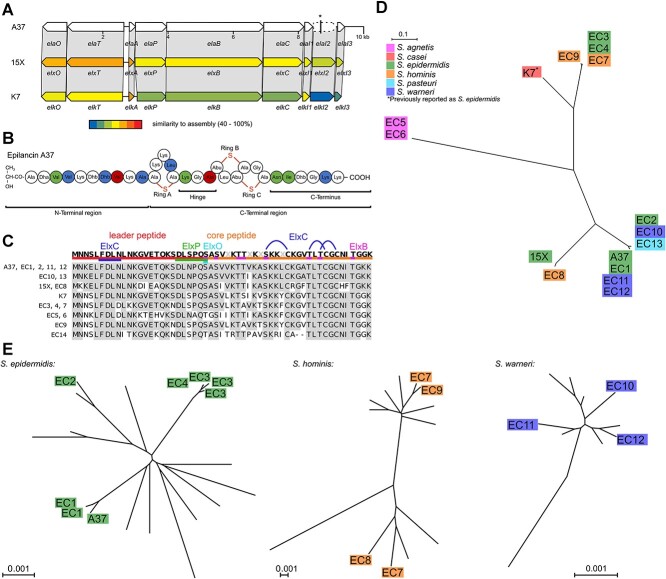
Analysis of A37 and the epilancin group. (A) Gene organization of the BGC of epilancin A37, compared with the BGCs of 15X and K7. The gene truncation of *ElaI2* is marked with an asterisk. (B) Predicted primary structure of A37. Blue: different from K7. Green: different from 15X. Red: different from K7 and 15X. Dha: dehydroalanine. Dhb: dehydrobutyrine. Abu: α-Aminobutyric acid. (C) Sequence alignment showing variations in the amino acid sequence of the epilancin scaffold for A37, 15X, K7, and all 14 ECs found in publicly available databases. Differences to the A37 sequence are highlighted in white. Leader peptide and interaction sites for posttranslationally modifying enzymes are illustrated on the consensus sequence. Assemblies with identical ECs were only included once in subsequent analyses. (D) Radial phylogram for BGC sequences of A37, 15X, K7, and EC1–13. (E) Radial species phylograms of all full assembly sequences containing an epilancin BGC available for *S. epidermidis*, *S. hominis,* and *S. warneri*. Additional assemblies not containing an epilancin BGC included to show the breadth and depth of the genetic diversity within each species. Versions of all phylograms with accession numbers of all assemblies used are shown in Fig. S1.

We investigated nasal swabs of healthy human volunteers and isolated the producer of an antibacterial compound directed against selected other colonies of the swab, which we identified as corynebacteria. This indicated the production of an antibiotic compound targeting specific members of the microbial community. Full-genome sequencing revealed the isolate to be a *S. epidermidis* strain harboring a biosynthetic gene cluster (BGC) with high similarity to previously described ECs K7 [16, 40] and 15X [17, 41] (Fig. 1A).

We termed the strain *S. epidermidis* A37 and the product, accordingly, epilancin A37. The publicly available sequence of the epilancin K7 operon was incomplete. We therefore fully sequenced and analyzed the genome of the K7 producer. Genomic analysis showed that the strain K7 belongs to the species *S. casei,* and not to *S. epidermidis* as previously reported [16].

The ~9.2-kbp epilancin BGC of *S. epidermidis* A37 consists of nine genes encoding the precursor peptide (*elaA*), a protease (*elaP*), a transporter protein (*elaT*), three tailoring enzymes (*elaBCO*) responsible for dehydration of serine and threonine into dehydroalanine and dehydrobutyrine (*elaB*), cyclization of dehydrated amino acids and cysteines to form lanthionine rings (*elaC*), and leader peptide hydrolysis and dehydrogenation of the N-terminal dehydroalanine to form lactate (*elaO*), and three putative immunity proteins (*elaI1*/*I2*/*I3*) [41]. All genes were conserved, and the encoded proteins showed 42%–82% sequence homology between the three strains A37, 15X, and K7. The putative immunity gene *elaI2* [41] is a pseudogene in *S. epidermidis* A37 because of the presence of frameshift mutation raising doubts regarding its relevance to provide producer immunity. However, as there is no experimental data available for the functions of *elaI1/I2/I3,* a more detailed evaluation of the impact of this mutation was not possible.

Multi-sequence alignment showed that the precursor peptide ElaA of A37 shares 82% similarity with its 15X and K7 counterparts (ElxA and ElkA). The core peptide of A37 shares 94% similarity with 15X and 87% similarity with K7. The ElxBCP recognition motifs are conserved across all three precursor peptides [40–43], suggesting that posttranslational modification is conserved between the epilancins. Based on this data, we predicted the structure of A37, which differs in multiple positions from both K7 and 15X (Fig. 1B). A37 features a 11-membered linear N-terminus, followed by a five-membered lanthionine ring, which is connected through a short three-membered hinge to a second ring system consisting of two intertwined four-membered lanthionine rings and a short six-membered C-terminus.

### Epilancins are prevalent in multiple staphylococci species with evidence of horizontal gene transfer

To investigate the distribution of epilancins among staphylococci, we searched all NCBI staphylococci-assemblies for BGCs containing a homolog of the A37 precursor peptide along with at least 50% of associated genes. We found a total of 18 additional assemblies containing 14 variants of the EC, isolated around the globe from diverse sources (Table S2). All ECs were found in coagulase-negative staphylococcal species: *S. epidermidis (EC1–4)*, *Staphylococcus agnetis (EC5–6), Staphylococcus hominis (EC7–9), Staphylococcus warneri (EC10–12)*, *Staphylococcus pasteuri* (EC13), and *Staphylococcus xylosus* (*EC14*). No ECs were found in any of the available *S. aureus*, *Staphylococcus capitis*, *Staphylococcus caprae*, or *Staphylococcus haemolyticus* assemblies.

Some of the ECs showed deviations in BGC composition (Fig. S2). EC1 has a premature stop codon in the putative immunity gene *elaI2*, similar to A37. In EC4, the dehydratase gene *elaB* is truncated. EC8 has the putative immunity gene *elaI3* in the reverse orientation. Three clusters are missing individual genes: EC5–6 lack the gene encoding the tailoring enzyme ElaO and have a premature stop codon in the gene for the transporter protein ElaT. EC14 is missing *elaOT*, *elaI2*, and *elaI3* and appears in the reversed orientation.

The precursor peptide has the same length in all strains except for EC14, which is two amino acids shorter. Individual differences in primary structure of the core peptide were observed, including notable variability in the N-terminal sequence that extended to substrate sites for dehydration by ElxB (Fig. 1C). In contrast, the substrates for Ring A formation and the full sequence of the Rings B–C region are fully conserved within all ECs, suggesting a key role of the C-terminal region for epilancin activity.

### Epilancin BGCs are most likely transferred across species boundaries

Whereas the genetic context of the 15X BGC is not publicly available, we found the BGCs of A37 and K7 to be located on a plasmid, suggesting that the gene cluster might be subject to horizontal gene transfer. To investigate this, we performed a phylogenetic analysis on the ECs (Fig. 1D). The BGCs did not form species-specific clades, which would have indicated isolated evolution of the clusters within the species. In contrast, various *S. epidermidis* clusters grouped with different *S. warneri* and *S. hominis* clusters. This pattern is consistent with multiple recent transfer events between different species. *Staphylococcus agnetis*-derived BGCs were distinct from the clusters found in other species, suggesting a distant transfer event in isolated evolution within the species.

To understand the genetic diversity of strains containing the BGCs, we calculated species-specific phylogenetic trees for *S. epidermidis*, *S. hominis,* and *S. warneri* (Fig. 1E) and included strains not containing an EC for reference. The species trees show the ECs in distant, unrelated clades. This further suggests that BGCs were transferred between strains by horizontal gene transfer and were not distributed by clonal expansion alone.

### A37 is particularly active against competitors of the producer strain in its ecological niche

To investigate A37 in more detail, we established a purification method for the peptide. The mass of the purified 30 amino acids long A37 peptide matched the bioinformatic prediction of 2984 Da (Fig. S2). Additionally, we observed the production of a likely methylated variant of A37 (2998 Da), which was always co-purified.

We assessed the antibacterial spectrum of purified A37 by determining the MIC against a range of model organisms and nasal isolates (Table 1). We observed a particular susceptibility of coagulase-negative staphylococci. Corynebacteria were exceptionally susceptible to A37 with MIC values of 0.5–2 μg/ml. The human commensal and important opportunistic pathogen *S. aureus* was not susceptible to A37. We found no evidence of A37 activity against Gram-negative species or mycobacteria.

**Table 1:** MIC of purified A37 against several bacterial species.

**Organism**	**MIC [μg/mL]**
*Corynebacterium accolens* 63VAs_B8	1
*Corynebacterium aurimucosum* 10VPs_Sm8	2
*Corynebacterium glutamicum* DSM 20300	1
*Corynebacterium kroppenstedtii* 82VAs_B6	0.5
*Corynebacterium propinquum* 63VAs_B4	1
*Corynebacterium pseudodiphtheriticum* 90VAs_B3	1
*Corynebacterium simulans* 81MNs_B1	2
*Corynebacterium striatum* 50MNs_Sm2	1
*Mycobacterium bovis* BCG	128
*Staphylococcus aureus* RN4220	>128
*Staphylococcus aureus* SA113	>128
*Staphylococcus aureus* COL	>128
*Staphylococcus aureus* USA300 JE2	>128
*Staphylococcus capitis* 15-B10536	4
*Staphylococcus epidermidis* ATCC12228	4
*Staphylococcus epidermidis* ATCC14990	16–32
*Staphylococcus sciuri* 1	8
*Staphylococcus simulans* 22	16–32
*Staphylococcus warneri* 15-O10013	4
*Bacillus subtilis* 168	16
*Escherichia coli* K12	>128

**Figure 2:**
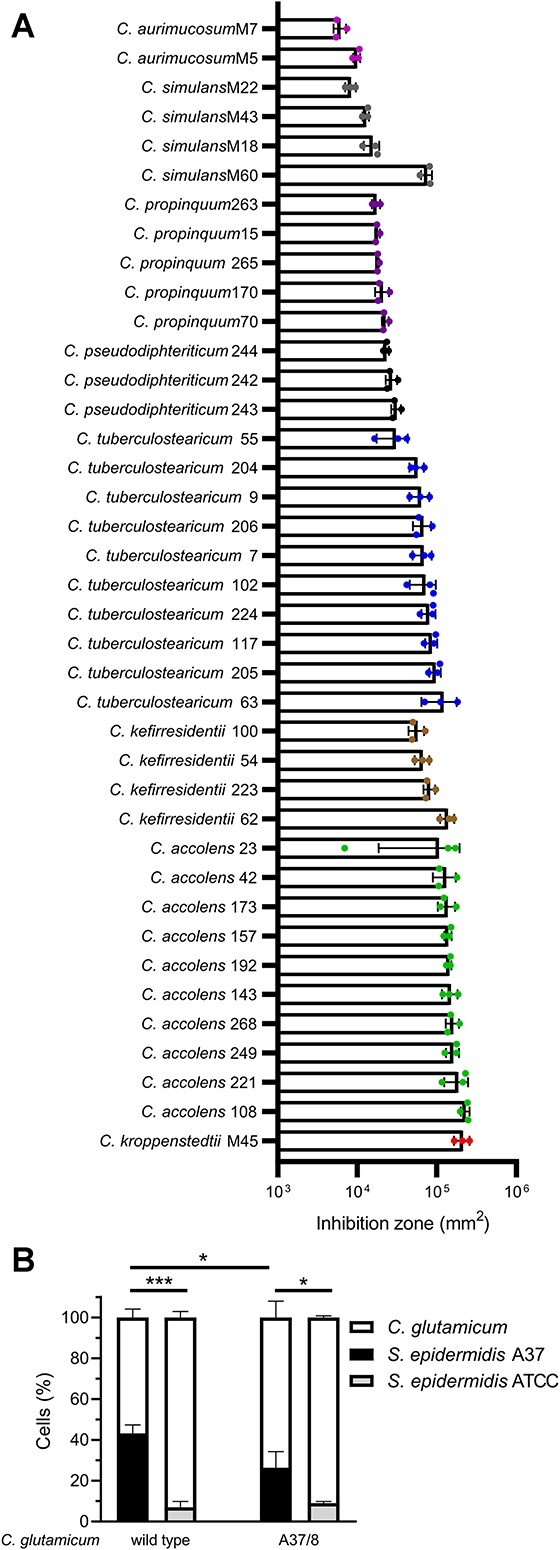
Antibacterial activity spectrum of A37. (A) Inhibition zones of *S. epidermidis* A37 against 39 nasal corynebacteria isolates. The inhibition zones were quantified by subtracting the spot area from the total inhibition area. Coloring corresponds to the different species of corynebacteria. Symbols show the values of three independent biological experiments. Bar graphs show mean ± SD. (B) Relative abundance of *S. epidermidis* and *C. glutamicum* cells after 3 h of co-incubation. White: *C. glutamicum*. Black: *S. Epidermidis* A37. Gray: *S. Epidermidis* ATCC 14990. Left: *C. glutamicum* wild type, right: *C. glutamicum* A37/8. Bar graphs show mean + SD of three independent biological experiments. Statistical significance was determined using unpaired two-tailed Students *t*-tests with a 95% confidence interval, *P* > .05; ^*^, *P* = .05–.01; ^***^, *P* = .001–.0001.

The high activity of purified A37 against various corynebacteria prompted us to further investigate the advantage of A37 production for *S. epidermidis* A37 against this genus. Within the nasal microbiome, both *S. epidermidis* and corynebacteria constitute major colonizers and competitors for resources in this hostile environment [2, 23, 44, 45]. To test for a putative ecological role of A37 production in this regard, we investigated the inhibitory capacity of *S. epidermidis* A37 against a total of 39 nasal isolates from eight different corynebacterial species. *Staphylococcus epidermidis* A37 showed inhibition zones against every strain of corynebacteria tested (Fig. 2A). Considerable species-related differences in the size of the inhibition zones were detected. We concluded that A37 production confers a significant benefit in the competition against corynebacterial species of the human nasal cavity. To quantify this effect, we challenged *S. epidermidis* A37 with the well-characterized *C. glutamicum* type strain DSM 20300 that was further used as a model organism. To this end, we mixed two cultures in a final OD-ratio of 1:99 (*S. epidermidis:C. glutamicum*). As a control, we prepared a mixture of the *S. epidermidis* type strain ATCC 14990 devoid of lantibiotic BGCs and *C. glutamicum* in the same ratio. After 3 h of co-incubation, the percentage of *S. epidermidis* cells in the mixture was assessed via widefield microscopy. *Staphylococcus epidermidis* A37 was clearly able to compete with *C. glutamicum* and established a substantial subpopulation constituting over 43% of all cells (Fig. 2B). In contrast, the type strain grew only slightly in relative abundance to 7%, showing a significant advantage of A37 production in the competition against *C. glutamicum*. To further confirm that this effect was mediated by epilancin production, we generated a *C. glutamicum* strain with an 8-fold reduction in A37 susceptibility by serial passaging named accordingly *C. glutamicum* A37/8 (Supplemental Information, Fig. S3A). We then repeated the experiment using this less-susceptible strain.

As expected, we did not observe a significant change in the competition with the *S. epidermidis* type-strain that does not produce an epilancin. In contrast, the ability of *S. epidermidis* A37 to displace the less-susceptible *C. glutamicum* variant was significantly reduced compared with the first experiment. This suggested that both the ability of *S. epidermidis* A37 to produce epilancin A37 and the susceptibility of the target strain are important for competition. Control single-strain growth curves (Fig. S3B) revealed that the growth dynamics of the strains did not add to, but rather opposed this correlation. This further adds to the significance of the observed competitive relevance of A37 production and resistance for producer and target.

### A37 enters the cytoplasm in a membrane potential-dependent manner without causing membrane disruption

Current knowledge about the MoA of epilancins is sparse. Early studies focused on the biosynthesis and structural chemistry of epilancins K7 and 15X. They were proposed to act by pore formation based on the structural similarity to nisin and recent work on 15X suggested a membrane disruptive effect against *Staphylococcus carnosus* as well as *B. subtilis* [8, 16–18, 21, 40, 41]. Given the wide distribution of epilancins in coagulase-negative staphylococci and their potential to affect the nasal microbial community, especially corynebacteria, we set out for an in-depth characterization of the cellular MoA of A37 against its natural target genus.

**Figure 3:**
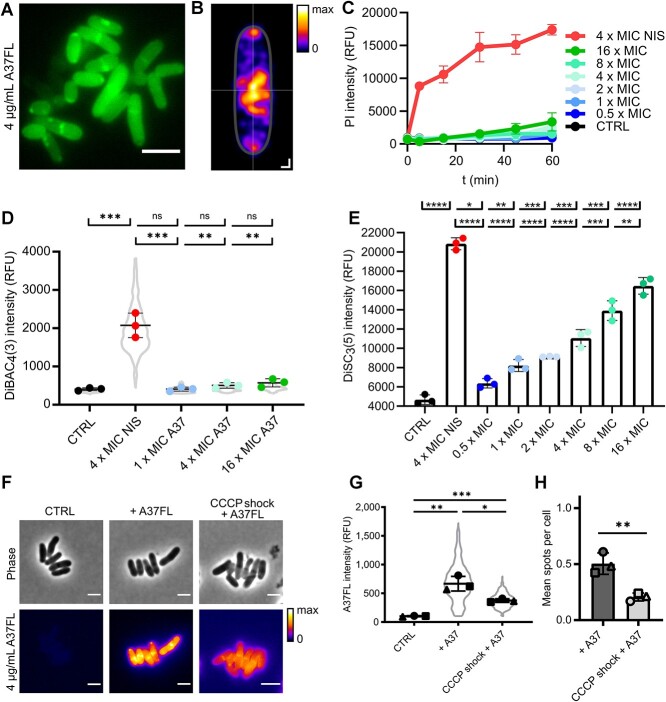
Characterization of A37 binding, impact on membrane function and uptake mechanism of A37. (A) Representative Airyscan super-resolution fluorescence micrograph of A37FL binding to *C. glutamicum* wild-type cells after 15 min of treatment. Scale bar, 2 μm. (B) Spatial distribution of A37FL fluorescence intensity maxima. Maxima were identified in *n* = 782 individual cells from three independent biological experiments. Scale bar, 0.2 μm for X and Y, respectively. (C) Fluorescence intensity of PI staining of *C. glutamicum* wild type over time during treatment with 0.5–16 x MIC A37, 4 x MIC nisin and an untreated control. Mean of three biological replicates ± SD. (D) DiBAC_4_(3) fluorescence intensities of cells of *C. glutamicum* wild type after 15 min of treatment with 4 x MIC nisin or 1–16 x MIC A37 and an untreated control. Mean of three biological replicates ± SD and cell data distribution. *N* ≥ 125 cells per replicate and condition. (E) DiSC_3_(5) release from cells of *C. glutamicum* wild type after 2 min of treatment with 4 x MIC nisin or 1–16 x MIC A37 and an untreated control. Mean of three biological replicates ± SD. (F) Representative micrographs of *C. glutamicum* wild type treated for 5 min with 10-μg/mL CCCP, followed by treatment with 4 x MIC A37FL 15 min, a control without CCCP treatment and an untreated control. Scale bar, 2 μm. (G) A37FL fluorescence intensities of individual cells. Mean of three biological replicates ± SD and cell data distribution. *N* ≥ 108 cells per replicate and condition. (H) Relative abundance of A37FL accumulation spots as mean spots per cell. Mean of three biological replicates ± SD from *n* ≥ 108 cells per replicate and condition. RFU: relative fluorescence unit. Statistical significance was determined using unpaired two-tailed Students *t*-tests with a 95% confidence interval, ns, not significant, *P* > .05; ^*^, *P* = .05–.01; ^**^, *P* = .01–.001; ^***^, *P* = .001–.0001; ^****^, *P* ≤ .0001.

To gain an understanding of its potential sites of action, we visualized the binding of the epilancin to live *C. glutamicum* cells in fluorescence microscopy using a derivative with BODIPY-FL-labeled lysine residues termed A37FL (Fig. 3). Using a mixture of 10% A37FL and 90% unlabeled A37, we unexpectedly found A37FL to localize primarily in the cytoplasm after 15 min of treatment, with no significant membranous signal detectable (Fig. 3A). Furthermore, distinct spots of accumulated A37FL were apparent. Analyzing the spatial distribution of these spots by determining their coordinates within the cells clearly illustrated that the spots were associated with the cell boundaries, preferentially at the cell poles and near the septum (Fig. 3B).

We assessed the effects of A37 on membrane potential and integrity over time and concentration in comparison to nisin. To quantify the impact of A37 on membrane integrity, we tested the effect of A37 on PI influx, a DNA stain which cannot pass intact cytoplasmic membranes (Fig. 3C). 4 x MIC nisin (16 μg/mL) served as positive control and immediately caused a massive increase in PI fluorescence because of its pore-forming activity. In contrast, A37 showed only negligible effects even after longer incubation times. A37 merely caused a slow, gradual lysis over time and only at high concentrations excluding pore formation and rapid membrane disruption as the primary MoA. This clearly demonstrated that the effect of A37 differed drastically from that of nisin. Furthermore, it corroborated the observation of cytoplasmic localization distinct from membrane disruptive lantibiotics and indicated that A37 penetrates the cytoplasmic membrane without inducing membrane damage (Fig. S4). The maintained integrity of the cytoplasmic membrane at MIC multiples for at least 1 h of treatment excluded that the cytoplasmic localization of A37 and the accompanied spot formation are an effect of cell death.

Membrane depolarization can be achieved without impairing membrane integrity. DiBAC_4_(3), an anionic dye, whose intracellular fluorescence intensity increases proportional to membrane depolarization [46] (Fig. S4) was used to quantify the impact of epilancin A37 on the transmembrane potential on the individual cell level. However, we observed no significant change in DiBAC_4_(3) signal after 15 min of treatment with up to 16 x MIC of A37 (Fig. 3D). In contrast, the treatment with 4 x MIC of nisin caused a drastic increase of intracellular DiBAC_4_(3) fluorescence. This showed that A37 had no depolarizing effect on *C. glutamicum* and further confirmed the differences between the MoAs of the two lantibiotics.

The bacterial transmembrane potential is a sum of multiple biophysical effects, which result in a net negative potential between the outside and inside of a bacterial cell. Accordingly, this potential attracts cations and can enable the intracellular aggregation of cationic molecules [47]. The polycationic nature of A37 combined with the observation that the epilancin had no impact on transmembrane potential homeostasis pointed to a putative uptake mechanism driven by the negative potential, causing the cytoplasmic localization of the epilancin. To investigate this hypothesis, release of the fluorescent dye DiSC_3_(5) was quantified during A37 treatment. DiSC_3_(5) is a cationic self-quenching dye that accumulates in cells proportional to their membrane polarization [46, 48, 49] (Fig. S4). Accordingly, release of the dye from cells results in de-quenching and fluorescence proportional to the amount of released dye. 4 x MIC nisin lead to massive and rapid release of DiSC_3_(5), in line with its membrane disrupting MoA (Fig. 3E). When cells of *C. glutamicum* were treated with A37, a rapid and dose-dependent release of DiSC_3_(5) was observed, reaching equilibrium within 2 min of treatment. The extent of this release was very moderate even at concentrations exceeding the MIC multiple times. Even 16 x MIC A37 still caused significantly less dye efflux than nisin, further underlining that impairment of membrane function is not the critical factor in the antimicrobial action of A37. As PI and DiBAC_4_(3) experiments demonstrated that A37 has no significant impact on membrane functionality, this efflux could not be explained by an A37-facilitated depolarization. Instead, it indicated a transmembrane-potential-driven uptake of the polycationic A37 into the cytoplasm, triggering a dose-dependent exchange of the cationic DiSC_3_(5) molecules with the polycationic A37 peptides (Fig. S4). To validate this, we treated cells with CCCP (10 μg/mL) prior to A37FL treatment, which is a protonophore that abolishes the transmembrane potential by transporting H^+^ cations across the cytoplasmic membrane. We observed a significant reduction in uptake of the epilancin, accompanied by a reduction in spot formation (Fig. 3F–H), proving that membrane potential plays an essential part in mediating the binding and translocation of A37 into the cytoplasm.

### A37 binds to and traverses giant unilamellar vesicle membranes *in vitro*

**Figure 4:**
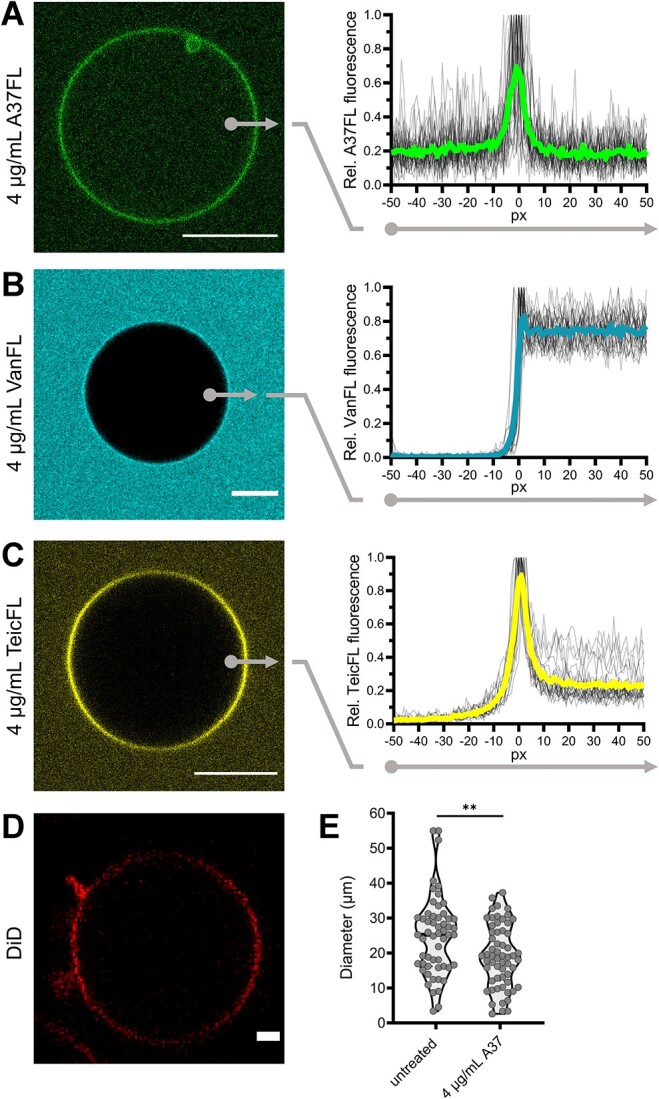
Interaction of A37FL with GUVs. (A–C) Treatment of DOPC/0.2-mol% DOPG GUVs with 4-μg/mL A37FL (A), vancomycin-FL (B), or teicoplanin-FL (C). Left: representative confocal micrographs of GUVs treated with either compound. Scale bar, 10 μm. Right: mean fluorescence intensities measured from 50 pixels (px) inside the GUV (px −50) through the GUV membrane (px 0) to 50 px outside the GUV (px 50). Mean of ≥24 individual measurements (thick line) and individual values (thin lines), plotted relative (Rel.) to the individual maximum value. (D) Representative confocal micrograph of DOPC/0.2-mol%PG GUVs treated with A37 and stained with DiD. Scale bar, 1 μm. (E) Diameters of GUVs treated with A37 and an untreated control. *N* ≥ 55 GUVs per condition. All individual measurements are shown. Statistical significance was determined using unpaired two-tailed Students *t*-tests with a 95% confidence interval, ^**^, *P* = .01–.001.

To understand the biophysical interaction of A37 with lipid bilayers, we investigated the binding of A37FL to giant unilamellar vesicles (GUVs), a well-established *in vitro* model for analysis of lantibiotic-membrane interactions. We observed moderate binding of A37FL to the GUV membranes and equal levels of A37FL fluorescence inside and outside of the GUVs within 15 min of treatment (Figs 4A and S5). To test for specificity of A37FL intra-GUV fluorescence, we additionally visualized interaction of the fluorescently labeled glycopeptide antibiotics vancomycin-FL and teicoplanin-FL with GUVs. As expected, vancomycin-FL did not bind to the GUV membrane (Fig. 4B), whereas the lipoglycopeptide teicoplanin-FL showed strong binding to the GUV membrane (Fig. 4C). In contrast to A37, we observed no fluorescence signal inside the GUVs for vancomycin-FL and teicoplanin-FL (Figs 4A–C and S5), showing that the glycopeptides were unable to penetrate the membrane layer. We thus concluded that the biophysical properties of A37 enable the peptide to specifically bind to and traverse GUV membranes. This supported our *in vivo* observations of A37 localizing to the cytoplasm without severe membrane disruption. In line with this, the interaction of the epilancin with the lipid bilayer had only moderate destabilizing impact on GUVs, as we observed blebbing of small vesicles and a general decrease in GUV size (Fig. 4 D and E). Still, GUVs overall stayed intact and we did not observe GUV collapse or heavy membrane deformation effects as previously described for nisin [12, 13].

### Formation of spots is essential for antibacterial activity

**Figure 5:**
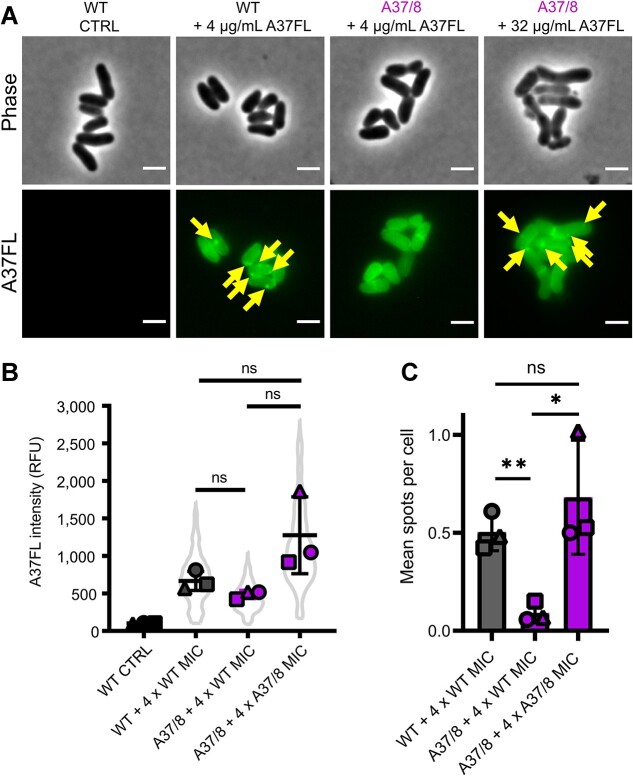
Analysis of the role of A37FL cytoplasmic localization and spot formation in antibacterial activity. (A) Representative micrographs of *C. glutamicum* wild type and *C. glutamicum* A37/8 treated with A37FL for 15 min and an untreated control. Left panel: untreated control. Middle panels: Cells of both strains treated with 4-μg/mL A37FL (corresponding to 4 x MIC of wild type and 0.5 x MIC of A37/8). Right panel: cells of *C. glutamicum* A37/8 treated with 32-μg/mL A37FL (corresponding to 4 x MIC of the strain). Arrows indicate A37FL accumulation spots. All images individually adjusted except for the negative control, which was adjusted to WT + 4 x WT MIC for clarity. Scale bar, 2 μm. (B) A37FL fluorescence intensities of individual cells. Mean of three biological replicates ± SD and cell data distribution. *N* ≥ 92 cells per replicate and condition. (C) Number of A37FL accumulation spots as mean spots per cell. Mean of three biological replicates ± SD from *n* ≥ 92 cells per replicate and condition. relative fluorescence unit. Statistical significance was determined using unpaired two-tailed Students *t*-tests with a 95% confidence interval, ns, not significant, *P* > .05; ^*^, *P* = .05–.01; ^**^, *P* = .01–.001.

Based on our *in vivo* MoA experiments, we reasoned that cytoplasmic localization and/or spot formation of A37 were crucial for antibacterial activity. To understand the relevance of intracellular spot formation *in vivo*, we quantified binding of A37FL to the *C. glutamicum* wild type and the serially passaged strain *C. glutamicum* A37/8. Despite the 8-fold difference in MIC, we observed no significant difference in the cytoplasmic concentration of A37FL when treated with 4-μg/ml A37FL (4-fold wild-type MIC). In contrast, a significant reduction in spot formation was apparent in A37/8 cells. Increasing the A37FL concentration to 32 μg/ml (4-fold MIC of strain A37/8) resulted in spot formation comparable to the effect of 4-fold MIC in the wild-type strain (Fig. 5). Together, this shows that it is not the cytoplasmic localization of A37, but the formation of spots that is central to its antimicrobial activity.

### A37 induces formation of intracellular membrane vesicles

**Figure 6:**
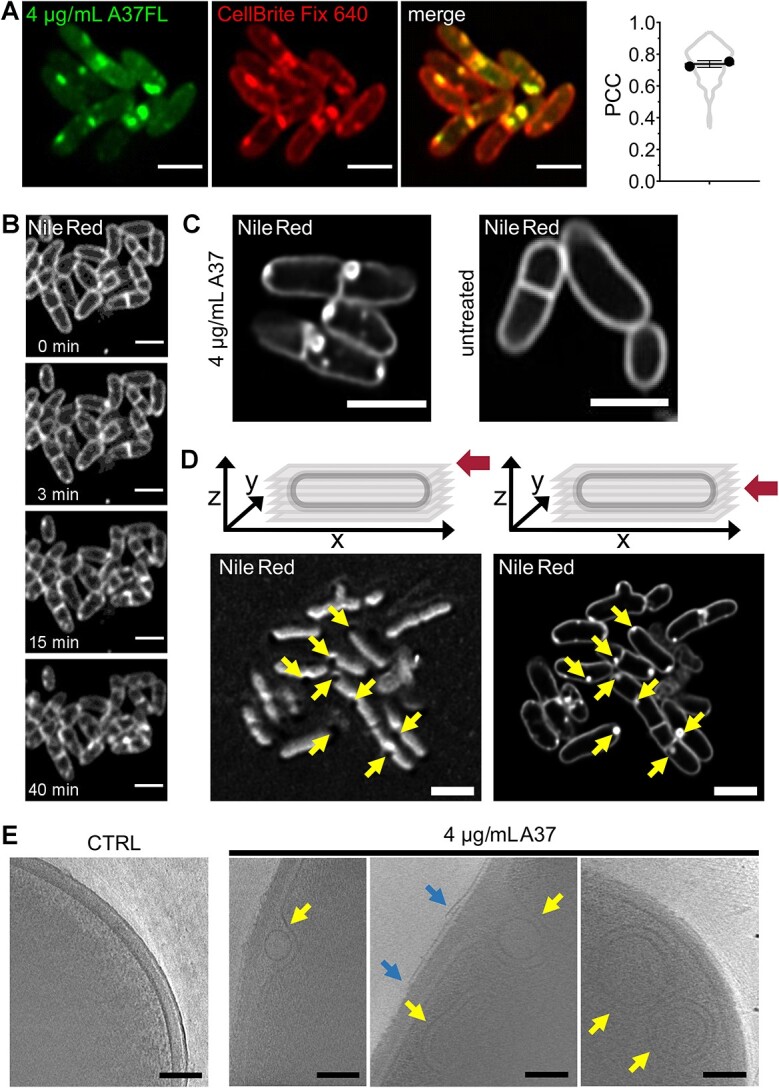
Intracellular vesicle formation by A37. (A) Left: representative AiryScan super-resolution micrographs of *C. glutamicum* wild type treated with 4 x MIC A37FL for 15 min and stained with the membrane dye CellBrite Fix 640. Right: colocalization of A37FL fluorescence and CellBrite Fix 640 fluorescence, determined as Pearson correlation coefficient (PCC) in individual cells. Mean of two biological replicates ± SD and cell data distribution. *N* ≥ 42 cells per replicate. (B) Representative timelapse of deconvolved AiryScan super-resolution micrographs of *C. glutamicum* wild type treated with 4 x MIC A37FL and stained with Nile red. (C) Representative deconvolved AiryScan super-resolution micrographs of *C. glutamicum* wild type treated with 4 x MIC A37FL for 15 min and an untreated control, stained with the membrane dye Nile red. (D) Deconvolved Z-Slices of AiryScan super-resolution micrographs of *C. glutamicum* wild type treated with 4 x MIC A37FL for 15 min and stained with the membrane dye Nile red. Schemes above the images illustrate the Z-positions. Yellow arrows indicate locations of intracellular vesicles and the corresponding membrane curvature. (E) Cryo-electron tomogrophic slices of *C. glutamicum* wild-type cells treated with 4 x MIC A37 and an untreated control. Yellow arrows indicate locations of intracellular vesicle formation. Blue arrows indicate locations of mycolic acid layer detachment. Scale bars A–D, 2 μm. Scale bar E, 100 nm.

To characterize the spots in detail, we performed a co-localization study of A37FL and the membrane dye CellBrite Fix 640 using Airyscan super-resolution microscopy (Fig. 6A). This visualized the presence of membrane dye patches inside the cell. Strikingly, A37FL spots co-localized with the patches. We suspected that A37 induces the formation of membrane vesicles, which protrude into the cytoplasm and are heavily loaded with the compound, either in the lipid layer or the inner compartment (Fig. 6A). To exclude artifacts of the A37FL fluorescence label, we also performed Airyscan super-resolution microscopy using native A37 and the bright and cell-penetrating dye Nile Red as a membrane stain. Live-cell microscopy with Nile Red visualized patch formation within the first few minutes of treatment (Fig. 6B). The high fluorescence intensity of Nile Red further enabled an efficient deconvolution. This confirmed the nature of membrane vesicles (Fig. 6C and D). Furthermore, Z-stack acquisition allowed to study the impact of A37 on membrane geometry. The cell membrane displayed a negative curvature at locations where the vesicles protruded into the cytoplasm (Fig. 6D). These effects were observed using conditions (4 x MIC and 15-min incubation time), that did not severely impair membrane integrity as demonstrated above. Finally, we used cryo-electron tomography to study the cellular effects of A37 at nanometer resolution. Cryo-electron tomograms of *C. glutamicum* cells treated with A37 revealed diverse features ranging from heteromorph membrane protrusions to circular unilamellar and double membrane vesicles (Figs 6E and S6). We likely captured an intermediate step in the formation of these vesicles, where membrane invaginations contain a single membrane vesicle. Additionally, we observed a detachment of the mycolic acid layer. None of these effects were observed in the untreated control (Fig. 6E).

## Discussion

Epilancins constitute a relatively scarcely studied group of lantibiotics. We here describe a member of this group produced by a nasal isolate of *S. epidermidis* and explore its ecological role and cellular MoA using bioinformatic, microbiological, biophysical, and microscopic techniques as well as cryo-electron tomography. We show that epilancins are widely distributed among staphylococci, convey major competitive advantage against corynebacterial competitors, and propose a comprehensive model for the mode of action for epilancin A37. First, the cationic peptide attaches to the cell surface because of charge attraction, as reported for numerous other cationic antimicrobial peptides [20, 50–56]. With its hydrophobic moieties A37 inserts into the membrane presumably via its C-terminus, which is very similar to the membrane-interacting part of nisin [14]. Subsequently, the peptide is translocated into the cytoplasm, in a process which is at least partially driven by the transmembrane potential. Conceptually, this adhesion and uptake process is similar to that of aminoglycosides [57]. Upon intracellular accumulation, the high local concentration of A37 enforces the formation of vesicles. We propose that the high cytoplasmic concentration of A37 stabilizes and enables the formation of these vesicles, possibly by self-interaction and interaction with anionic membrane components. The continuous aggregation of membrane components in the vesicles leads to cell death, as the connection of vesicle formation and antimicrobial activity shows. This may involve trapping of essential proteins and/or detrimental changes in lipid distribution. Consequently, this would lead to a lethal collapse of membrane bound biosynthetic machineries and core metabolic processes similar to the recently described mechanism of the structurally unrelated compound rhodomyrtone [58]. The detachment of the mycolic acid membrane likely contributes to cell death.

To the best of our knowledge, this is a hitherto not described MoA for lantibiotics. The MoA of many lantibiotics is entirely unknown and nearly all of those with a characterized MoA kill via pore-formation or inhibition of peptidoglycan biosynthesis [5–7]. In the case of the well-studied nisin, the mechanism of pore-forming activity has been elucidated down to the structural level and is based on the interaction of the N-terminus with Lipid II [8–10, 12, 14]. The MoA of epilancins was initially thought to involve pore-formation because of the structural similarity to the nisin C-terminus [20, 40]. Observed DiSC_3_(5) efflux seemed to agree with this hypothesis [18, 19], as it is often caused by membrane depolarization [46]. However, our findings of intracellular aggregation of the polycationic A37 contextualize these results, showing that DiSC_3_(5) efflux is rather caused by the concentration dependent cation exchange with the epilancin. In line with this, we could show that the net transmembrane potential is indeed maintained, as the anionic dye DiBAC_4_(3) proved unable to bind to cells. This could also apply to the observed DiSC_3_(5) efflux for other epilancins in other organisms [18, 19]. However, this should be carefully evaluated, since it has been suggested before that the specifics of epilancin MoA differ depending on species membrane composition [19, 21]. In this regard, the cell envelope structure of corynebacteria, which is characterized by a mycolic acid-bound arabinogalactan layer and the presence of mycolic acid precursors in the corynebacterial membrane, might modulate the epilancin MoA and activity against this genus. Furthermore, conflicting data on the ability to disrupt liposomes and effects on membrane integrity [8, 18, 19, 21] made it difficult to propose a general MoA for epilancins. Our data agree with the hypothesis that epilancin MoA depends on the lanthipeptide sequence and target specifics, because we could not find any indications of severe membrane disruption in A37-treated corynebacteria. 15X was recently found to induce membrane depolarization in *B. subtilis* and *S. carnosus* without cytoplasmic internalization [19, 21]. This illustrates the importance of analyzing the MoA of these lantibiotics in the context of interest, e.g., their ecological role (in this work) or therapeutic potential against pathogens (in previous works). Epilancin-mediated membrane disruption may be specific against staphylococci and membrane vesicle formation specific against corynebacteria, whereas other MoAs may eventually be proposed for other species. From an ecological perspective, a fully generalized MoA may not be feasible given that there are many species beside staphylococci and corynebacteria in the nose and skin microbiota. However, a general basis for antimicrobial activity may be proposed, which is constituted by the amphiphilic cationic nature of the peptides and their ability to interact with lipid membranes. On top, evolutionary tailoring of the scaffold may allow for a competitor-specific modulation of that membrane interaction. In this regard, conservation of the Lan-ring systems and overall scaffold might provide the basis for membrane interaction, and sequence variation of N-terminus and hinge region may be important for adaption of the activity. To this end, removal of the N-terminus from 15X was shown to significantly reduce its antibacterial activity [41], and the hinge region sequence of nisin was shown to be important for modulating its membrane disruptive activity [14, 59–61].

*In vitro*, A37 binds to and transverses membranes. We propose that this interaction is governed by the C-terminal region based on its similarity to that of nisin. For nisin, it has been shown that this region, composed of three-ring systems and a hinge, spans the cytoplasmic membrane and is critical for modulating activity [14]. The flexible C-terminus was shown to reach into the cytoplasm [14], illustrating the amphiphilic nature of this region, which in case of epilancins might enable dissociation from the membrane. Our *in vitro* observation differed from the *in vivo* results in two key aspects: (i) we did not observe intra-GUV accumulation of A37FL. This corroborates the hypothesis of a transmembrane-potential-driven accumulation *in vivo*. (ii) A37FL did not lead to notable formation of vesicles from GUV membranes. *In vivo*, the effect of vesicle formation and A37 aggregation within or at the formed vesicles may be caused by the peptides’ high intracellular concentration because of accumulation and/or by interaction with membrane components absent in the *in vitro* setup (e.g. certain lipid species, proteins). The MoAs of the structurally related lantibiotics Pep5 and epicidin 280 [4] have not been resolved so far [8, 62–65].

The wide distribution of epilancins within staphylococci strongly suggests a high ecological relevance. This is corroborated by the abundance of the scaffold in different coagulase-negative staphylococci isolated from different ecological niches. In this regard, primary and potentially secondary structural diversity indicates optimization of the scaffold to the particular competitors. A37 seems to be optimized for the specific composition of corynebacterial membranes, given the extraordinary activity against members of this genus. Staphylococci and corynebacteria constitute key genera of the human nose and skin microbiota closely associated with health and disease [2, 23–25]. The ubiquitous activity against nasal corynebacteria suggests a crucial role of the epilancin to drastically shape the nasal microbiome for the advantage of the producer. The need to produce such a compound indicates pronounced interspecies competition specifically between coagulase-negative staphylococci and corynebacteria, in line with previous reports on nasal microbiome composition [23, 44]. Our work sheds light on the importance and MoA of epilancins in this “bacterial warfare.”

## Supplementary Material

2024-02-23_final_SI_final_wrae044

## Data Availability

All data needed to evaluate the conclusions in the paper are present in the paper and/or the Supplementary Information.
