# Supplementary material for: Staphylococcus epidermidis bacteriocin A37 kills natural competitors with a unique mechanism of action
Source: ISME J. 2024 Mar 12;18(1):wrae044. doi: 10.1093/ismejo/wrae044 (PMC10988021; doi:10.1093/ismejo/wrae044)
Supplement: 2024-02-23_final_SI_final_wrae044 [file 2024-02-23_final_si_final_wrae044.pdf]

Supplementary Information for

Staphylococcus epidermidis bacteriocin A37 kills natural competitors with a unique mechanism of action

Jan-Samuel Puls, Benjamin Winnerling, Jeffrey John Power, Annika Marie Krüger, Dominik Brajtenbach, Matthew Johnson, Kevser Bilici, Laura Camus, Thomas Fließwasser, Tanja Schneider, Hans-Georg Sahl, Debnath Ghosal, Ulrich Kubitscheck, Simon Heilbronner, Fabian Grein

Corresponding author: Fabian Grein
Email: grein@uni-bonn.de

This PDF file includes:

Supplementary text
Figures S1 to S6
Tables S1 to S4

Supplementary text

Isolation and purification of epilancin A37

For the production of epilancin A37, standard LB medium was inoculated with a *S. epidermidis* A37 preculture at 1% (v/v) and incubated overnight (37°C, 120 rpm). Cells were removed by centrifugation (9,500 rpm, 25 min) and the cleared supernatant was filter-sterilized (0.2 µm pore size). The sterile supernatant was incubated with Sepabeads SP-207 (1% (w/v), 2 h, 20°C, 120 rpm; Sigma-Aldrich, US). The Sepabeads were transferred into a chromatography column with frit and washed with methanol in increasing concentrations (0/30/70% MeOH in water, 5 column volumes), followed by elution of bound A37 with HCl-acidified methanol (0.125% (v/v) 12 M HCl). After recovery, A37 was purified by anion exchange FPLC (HiTrap SP HP; Cytiva, US) and a two-step reversed phase HPLC program (Multokrom 100 10 C18/MultoHigh-Bio-300-5 C4) with an acetonitrile gradient elution. Between the chromatography steps, spot assays of the elution fractions were conducted to identify fractions containing antimicrobial activity for further processing. Lastly, acetonitrile was removed in a rotary evaporator before samples were freeze-dried and stored at -20°C. Lyophilized A37 was resuspended prior to use in water or buffer depending on experimental requirements.

Whole genome sequencing

For short read sequencing, DNA was isolated from cell pellets using DNeasy PowerSoil Pro Kit (QIAGEN, NL) according to the manual's instructions with 2 minutes of vortexing in PowerBead Pro tubes. Libraries were prepared using the DNA Prep (M) Tagmentation kit (Illumina, US) according to the manufacturer's protocol with 500 ng DNA input and 5 cycles indexing PCR. The libraries were checked for correct fragment length on a 2100 BioAnalyzer (Agilent, US) and pooled equimolarly. The pool was sequenced on an MiSeq v3 600 cycles Flow Cell (Illumina) with 2 x 150 bp read length and a depth 70x genome coverage. For long read sequencing, the cell pellet was resuspended in 600 µL ATL buffer (Qiagen #939011) and transferred to a ZR BashingBead Lysis Tube (Zymo Research #S6012-50). The tube was vortexed horizontally for 2 minutes on a vortex shaker. To optimize the DNA extraction, the supernatant was taken off and digested with RNAse A (Qiagen). The DNA was then automatically purified with the QIAamp 96 QIAcube HT kit (Qiagen #51331) with additional proteinase K on a QIAcube HT following the manufacturer's instructions. For library generation, the Ligation Sequencing Kit (LSK) 109 (Oxford Nanopore, UK) was used with native barcoding, following the manufacturer's protocol, with 500 ng DNA input per sample and prolongated incubation times. Library size was assessed on a FEMTO Pulse (Agilent) and libraries were pooled equimolarly before sequencing on a FLO-PRO002 flow cell on a Nanopore PromethION device. 14.5 million reads with 55 Gb were generated. Libraries were created using the Nextera tagmentation kit (Illumina) and sequenced on a MiSeq NextSeq > add long reads with paired-end sequencing (2 x 241 bp). DNA sequence reads were assembled using nf-core pipeline bacass (v2.0.0 [1]). Assembled scaffolds were annotated using PGAP (NCBI, v.2021-11-29.build5742 [2]) and curated using NCBI Genome Workbench (v3.7.0[3]). Default antiSMASH 5.0 settings were used to detect biosynthesis gene clusters.

Microscopic methods

Labeling of A37 with BODIPY-FL

Lysine residues of A37 were labeled with an amine-reactive Bodipy-FL NHS Ester (Thermo Fisher, US). A37 was solved in DMF to a final concentration of 5 mg/mL. BODIPY-FL-NHS-Ester was solved in DMSO to a final concentration of 10 mg/mL. Both solutions were mixed molar ratio of 1:3 (A37:BODIPY-FL). The mixture was vortexed for 2 h at room temperature and purified via cation exchange FPLC using a Bio-Rad NGC Chromatography System equipped with a HiTrap SP HF 1 mL column. This enabled elution of purified A37FL and separation of overly labelled A37FL (with multiple labelled lysine residues) through the reduction in cationic charge. The antimicrobial activity of the labeled compound was confirmed with spot assays against *C. glutamicum* wild type. After determining the concentration of A37 in active samples, MICs of the purified A37FL against *C. glutamicum* DSM 20300 were determined. MICs of A37FL were identical to unlabeled A37.

Labeling of Teicoplanin with FITC

Teicoplanin was labeled with FITC (Thermo Fisher, US) by solving both reagents in 100 mM carbonate buffered saline, pH 9.0 in a w/w ratio of 1:1, followed by 12 h of incubation at 4°C under constant stirring. Unbound FITC was removed by three rounds of 24 h dialysis in 10 mM PBS, pH 7.4 at 4°C and verified via TLC.

A37FL widefield microscopy

Cultures were grown as stated above until they reached OD_{600}=0.5 and then incubated for 15 min at 37°C with the appropriate concentration of A37FL. Cultures were washed three times in medium (2 min, 13,000 rpm), mounted on 1% agarose slides and A37FL fluorescence was analysed microscopically. If abolishment of the membrane potential was required, cultures were incubated for 5 min at 37°C with 16 µg/mL CCCP and washed three times in medium prior to treatment with A37FL. For all experiments, a mixture of 10% A37FL with 90% unlabeled A37 was used.
Widefield fluorescence microscopy of A37FL and $DiBAC_4(3)$ was performed on a Carl Zeiss AxioObserver Z1 equipped with a HXP 120 C lamp, a Carl Zeiss αPlan-APOCHROMAT 100×/1.46 oil immersion objective, and a Carl Zeiss AxioCam MRm camera (all Carl Zeiss, Germany). The setup was controlled using the Carl Zeiss Zen Blue 2.0 Software. Visualization was achieved using Carl Zeiss filter set 38 (450–490 nm excitation wavelength, 495 nm beam splitter and 500–550 nm emission wavelength).

GUV microscopy

Giant unilamellar vesicles (GUVs) were prepared via electroformation out of 1,2-dioleoyl-sn-glycero-3-phosphocholine (DOPC) and 0,2mol% 1,2-dioleoyl-sn-glycero-3-phospho-(1'-rac-glycerol) (DOPG). Required lipids were solved in 1.3 mM chloroform. Electroformation was performed on cleaned indiumtinoxide (ITO) coated glass coverslips (SPI Supplies, US). Cleaning of the coverslips was accomplished by sonication in 0.1 M sodium hydroxide for 15 minutes followed by subsequent washing with H_2O dest. and acetone. Two cleaned coverslips were connected via copper band attached to the conductive sides, silicone-coated O-rings were mounted on the coverslips and 25 µL of lipids were filled into the O-rings. 275 µL of 250 mM sucrose were added after chloroform evaporation and O-rings were covered with another set of connected coverslips. GUVs were formed with an alternating voltage of 10 V/12 Hz for 4 h at room temperature provided by a pulse generator attached to the copper bands. The GUV solution was then transferred to 1 mL of 250 mM glucose solution in a 35-mm-µ-Ibidi plate (Ibidi, Germany). Depending on the experiment, the 4 µg/mL of A37 or A37FL or vancomycin-FL or teicoplanin-FL and/or a 1:200 Vybrant DiD Cell-Labeling Solution (Thermo Fisher, US) were added to the glucose solution beforehand. For all experiments, A37FL, vancomycin-FL and teicoplanin-FL were used as mixtures of 10% labeled and 90% unlabeled compound.
Imaging was performed with a confocal laser scanning microscope (LSM 880, Carl Zeiss, Germany) equipped with an α-Plan-Apochromat 63x/1.46 Oil Corr M27 objective (Carl Zeiss, Germany). Bodipy-FL and DiD were excited with an Argon-Laser at a wavelength of 488 nm and 514 nm, respectively.

Confocal Airyscan super-resolution microscopy

For microscopical imaging, a culture of C. glutamicum wild type was grown in MH medium at 37°C under constant shaking until it reached OD¬600=0.5. Cells were then incubated for 15 min at 37°C with the appropriate concentration of A37, or A37FL respectively, washed three times with MH medium, and mounted on 1% agarose slides. For all experiments with A37FL, a mixture of 10% A37FL with 90% unlabeled A37 was used. For timelapse microscopy, cells were mounted on 1% agarose slides immediately prior to addition of A37 and imaged at 37°C during treatment with A37. If needed, a membrane dye was added onto the agarose gel (1:1000 CellBrite Fix Red; Biotium, US, or 20 nM Nile Red; Thermo Fisher, US, respectively). If pre-incubation with the membrane dye was required for the experiment, the dye was added to the cells for 5 min and washed off three times with MH medium before addition of A37. In that case, no membrane dye was added to the agarose gel after incubation with A37.
A Zeiss LSM 880 microscope with an Airyscan detector (Carl Zeiss, Germany) was used for acquisition of confocal images. Z-Stacks were taken with one of two 63x oil immersion objectives (Plan-Apochomat 63x/1.4 Oil or αPlan-Apochromat 63x/1.46 Oil). Fluorescence excitation was accomplished using 633 nm

(CellBrite Fix 640), 561 nm (Nile Red), 488 nm (A37FL) and 405 nm (DAPI) emitting lasers. Additional emission filters (BP 495 – 535 nm + LP 555 nm) were used for acquisition of A37FL labeled cells. Deconvolution of raw confocal Airyscan data was performed with Huygens Professional (Huygens compute engine 21.04.0p2 64b) software with the fast Classic MLE deconvolution algorithm in a maximum of 40 iterations with a desired signal to noise ratio of 12 in optimized iteration mode. Not deconvolved images were 3D-Airyscan processed with Carl Zeiss ZEN (ZEN 2.3 SP1 FP1 (black)) software.

Cryo-electron tomography sample preparation and data processing

C. glutamicum cells were grown to exponential phase (OD600 of 0.5) in MHB. This culture was incubated with either a buffer control or 4 µg/mL A37 antimicrobial peptide for 15 min at 37 ºC shaking at 120 rpm. After incubation, the cells were precipitate by centrifugation at 10000 rcf for 2 minutes. Cells were resuspended to their original volume in MHB, this process was repeated 2 more times. On final resuspension, the cells were concentrated 5 times, a 20 µL aliquot of the cells was mixed with 4 µl of 10 nm colloidal gold beads (Sigma-Aldrich) precoated with BSA. Samples were quickly applied onto glow-discharged copper R2/2 200 Quantifoil holey carbon grids (Quantifoil Micro Tools, Germany). The grids were then blotted and plunge-frozen in a liquid ethane using an FEI Vitrobot Mark IV (blot force 8, blot time 4 seconds) and stored in liquid nitrogen for imaging. Tilt series were recorded of frozen C. glutamicum cells in a Titan Krios G4 300 keV field emission gun transmission electron microscope (Thermo Fisher, US) equipped with a Gatan imaging filter and a K3 Summit direct detector in counting mode (Gatan, US), using the FEI Tomography software 5 (FEI Company, US) and a total dose of ~130 e/A2 per tilt series and a target defocus of 8 µm under focus. The magnification of 26,000 x mag, and pixel size of 3.4 Å. Energy-filtered tilt series of images of the cells were automatically collected from 0° to +/−45° at 2° intervals using a dose symmetric scheme [4]. Images were aligned using gold fiducial markers in eTomo software. Simultaneous Iterative Reconstructions were produced using TOMO3D [5] and images were generated using IMOD [6, 7] .

Image data analysis

Image Analysis was performed using Fiji (ImageJ) version 2.0.0-rc-69/1.52p; Java 1.8.0_172 (64 bit) [7] and the ImageJ Plug-In MicrobeJ version 5.13l (20)–beta [8]. Statistical analysis was performed using GraphPad Prism 8.0.2 (263). Individual cell values or population mean values were plotted either as superplots or in another appropriate way to increase data intelligibility whenever applicable [9]. If needed for visualization of mean values and single cell data distribution and not stated otherwise, violin plots were generated from data sets after exclusion of outliers using Prism 8 robust regression and outlier removal (ROUT) with Q = 1% [10] for improved visualization clarity of the mean values. Outlier exclusion did not impact the data analysis or the conclusions drawn from data in any way and was done purely for visualization purposes.

Fluorescence intensities and Pearson correlation coefficient were determined as mean per cell using MicrobeJ. To quantify the spots of A37 in cells of C. glutamicum, the sum of visible spots in all cells of a sample was determined after individual brightness and contrast adjustment for each single cell image. The mean number of spots per cell was calculated as the ratio of the sum of spots and the number of cells. Fluorescence maxima were determined on the individual cell level using the MicrobeJ maxima detection process. Maxima were defined as pixels (px) of highest local fluorescence (MicrobeJ “point” function). Maxima detection was limited to five A37FL maxima per cell to ensure that only the maxima with highest fluorescence intensities relative to the cell mean intensity were detected (MicrobeJ “CutOff” function). Lower threshold (MicrobeJ “tolerance” function) was set to 10% of the population mean intensity to detect maxima even in cells with comparably low intensity and exclude potential artifacts and outliers without significant fluorescence intensity. Density of maxima was calculated and visualized using MicrobeJ.

GUV section fluorescence intensities were quantified as the mean width intensity of a 10 px wide and 101 px long rectangle with the 51st px placed on the GUV membrane. Mean fluorescence intensities inside and outside of GUVs was quantified by measuring the mean fluorescence intensities of 50 x 50 px squares randomly distributed within or outside GUV boundaries. GUV size was determined using FIJI measurement tools.

Resistance acquisition of *C. glutamicum* A37/8

We performed whole genome sequencing of *C. glutamicum* A37/8 and found a total of seven single nucleotide polymorphisms compared to the parent strain *C. glutamicum* DSM 20300 (Table S3). None of these were found genes encoding characterized proteins of *C. glutamicum*, thus preventing a hypothesis regarding the impact on the strain's physiology and possible reasons for its decreased susceptibility towards A37.

We tested the antimicrobial impact of several cationic antimicrobial substances against *C. glutamicum* A37/8 and observed no notable change in MICs compared to the parent strain (Table S4), indicating a specific resistance mechanism against A37. The fact, that uptake of A37 remains functional, but vesicle formation is decreased suggests, that this mechanism is directed against the vesicle formation (Fig. 5).

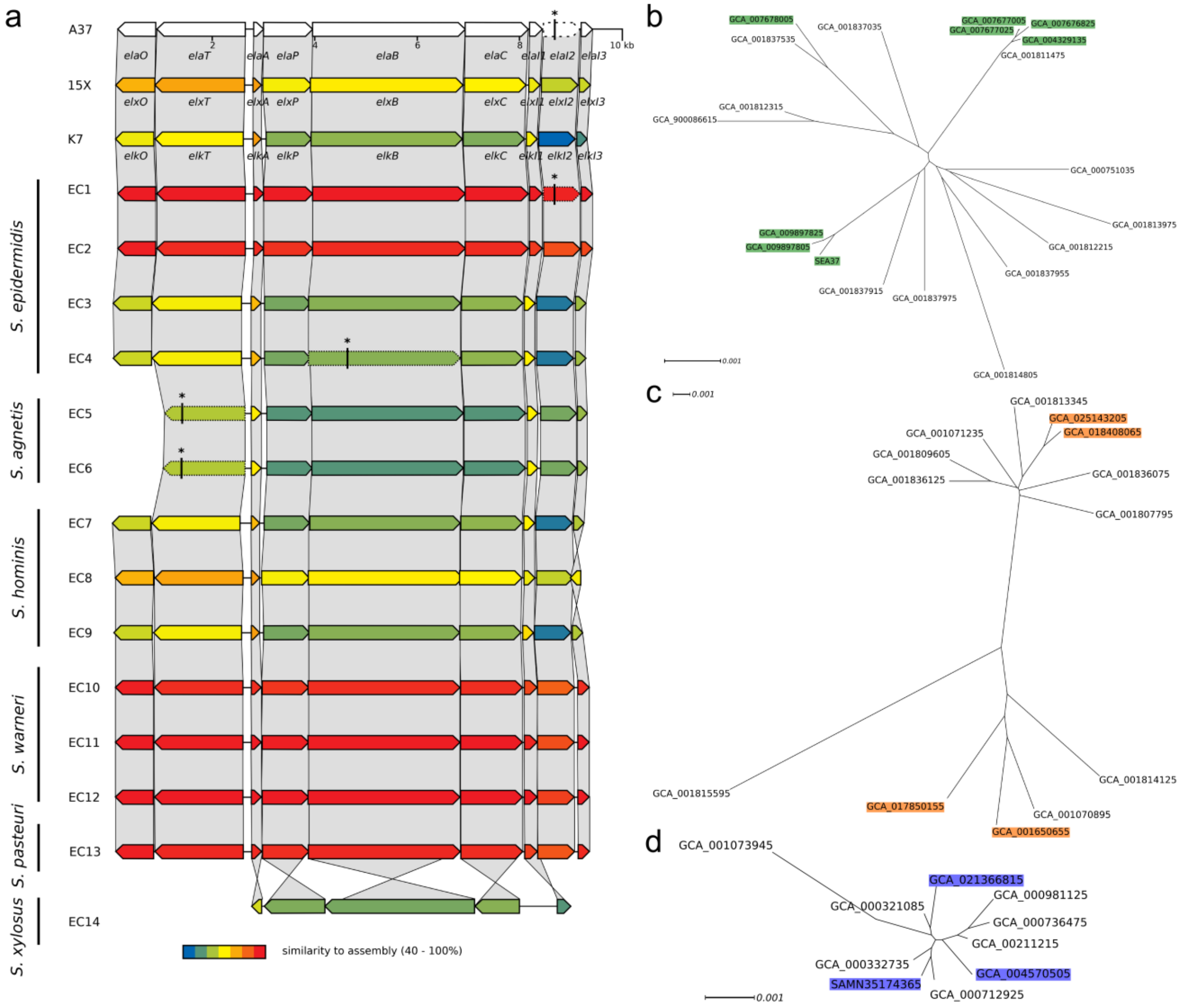

Fig. S1. a) Sequence similarity comparison of all epilancin BGCs. Gene truncations are marked with an asterisk. b) – d) Radial species phylograms from Fig. 1e with gene accession numbers (GCAs) shown. b) *S. epidermidis*. c) *S. hominis*. d) *S. warneri*.

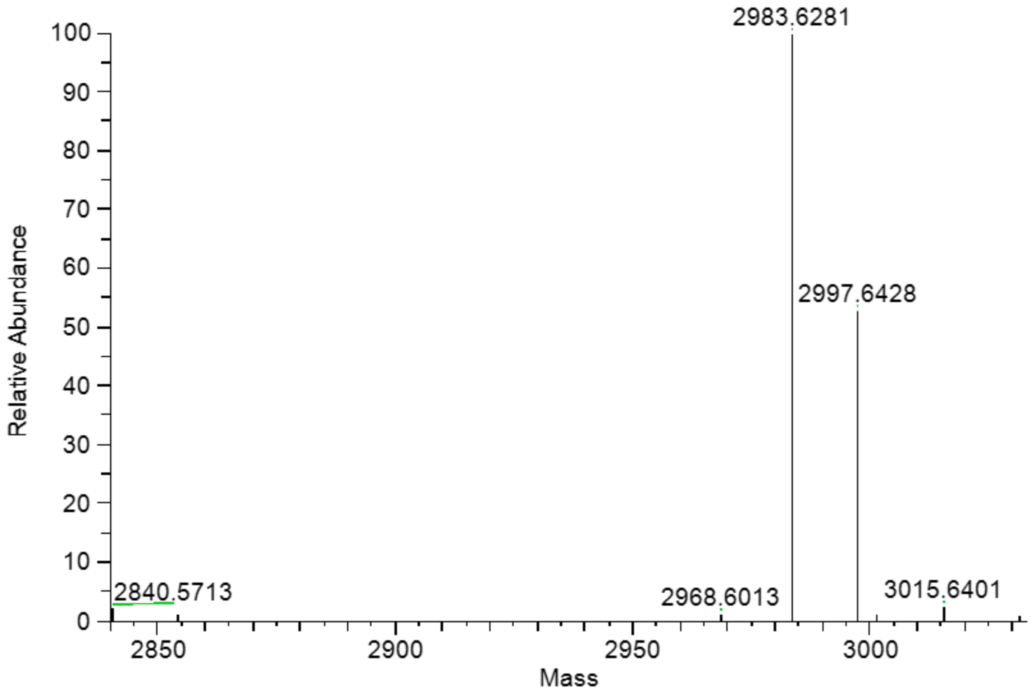

Fig. S2. Mass spectrometry analysis of A37 solution after HPLC purification. Representative deconvoluted spectrum of neutral monoisotopic masses of purified antimicrobial peptide A37 analyzed by ESI-MS (ThermoScientific Orbitrap Velos), expressed as relative abundances.

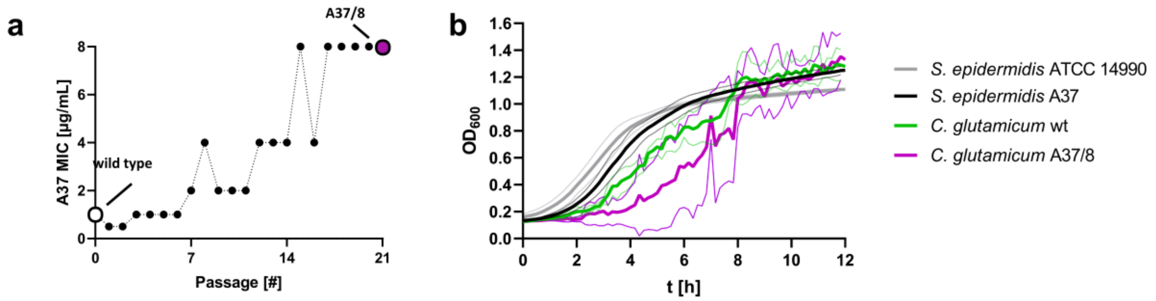

Fig. S3. a) Development of the A37 MIC of *C. glutamicum* DSM 20300 during serial passaging. b) Growth curves of *S. epidermidis* ATCC 14990, *S. epidermidis* A37, *C. glutamicum* wild type and *C. glutamicum* A37/8. Lines show the mean of three independent biological experiments ± SD.

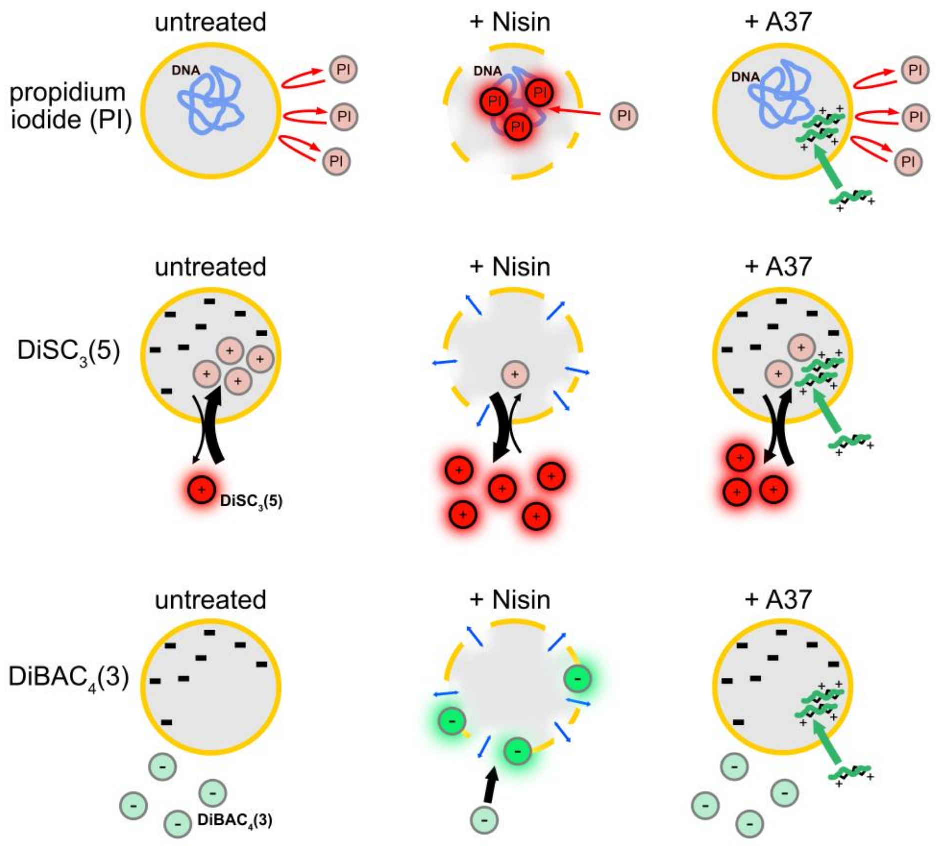

Fig. S4. Schematic illustration of the mechanisms of propidium iodide, $DiSC_3(5)$ and $DiBAC_4(3)$ fluorescence behaviour and how nisin and A37 modulate fluorescence of the dyes.

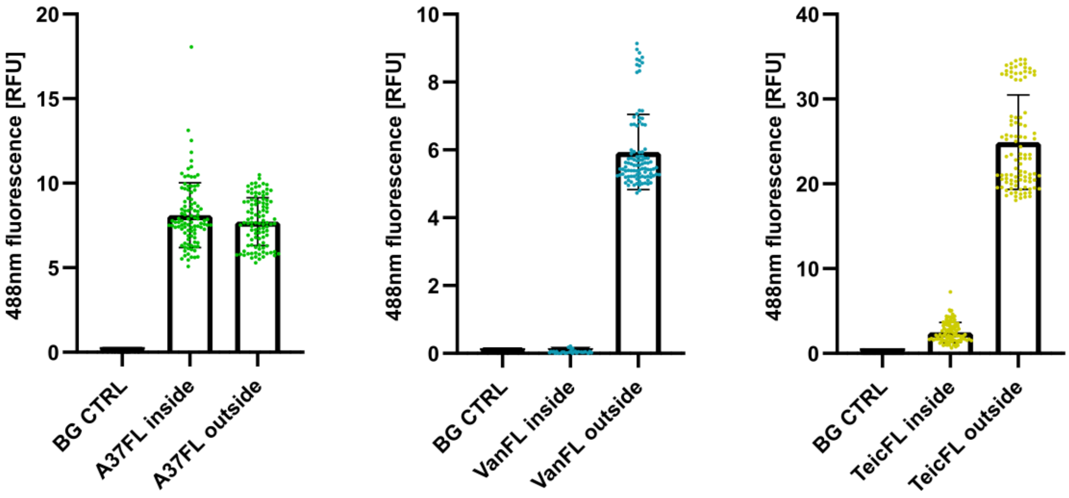

Fig. S5. Mean fluorescence intensities of 50 x 50 px squares inside and outside of GUVs treated with A37FL (left), vancomycin-FL (middle) and teicoplanin-FL (right). BG CTRL: Background control, GUV medium imaged at identical experimental conditions without a fluorophore.

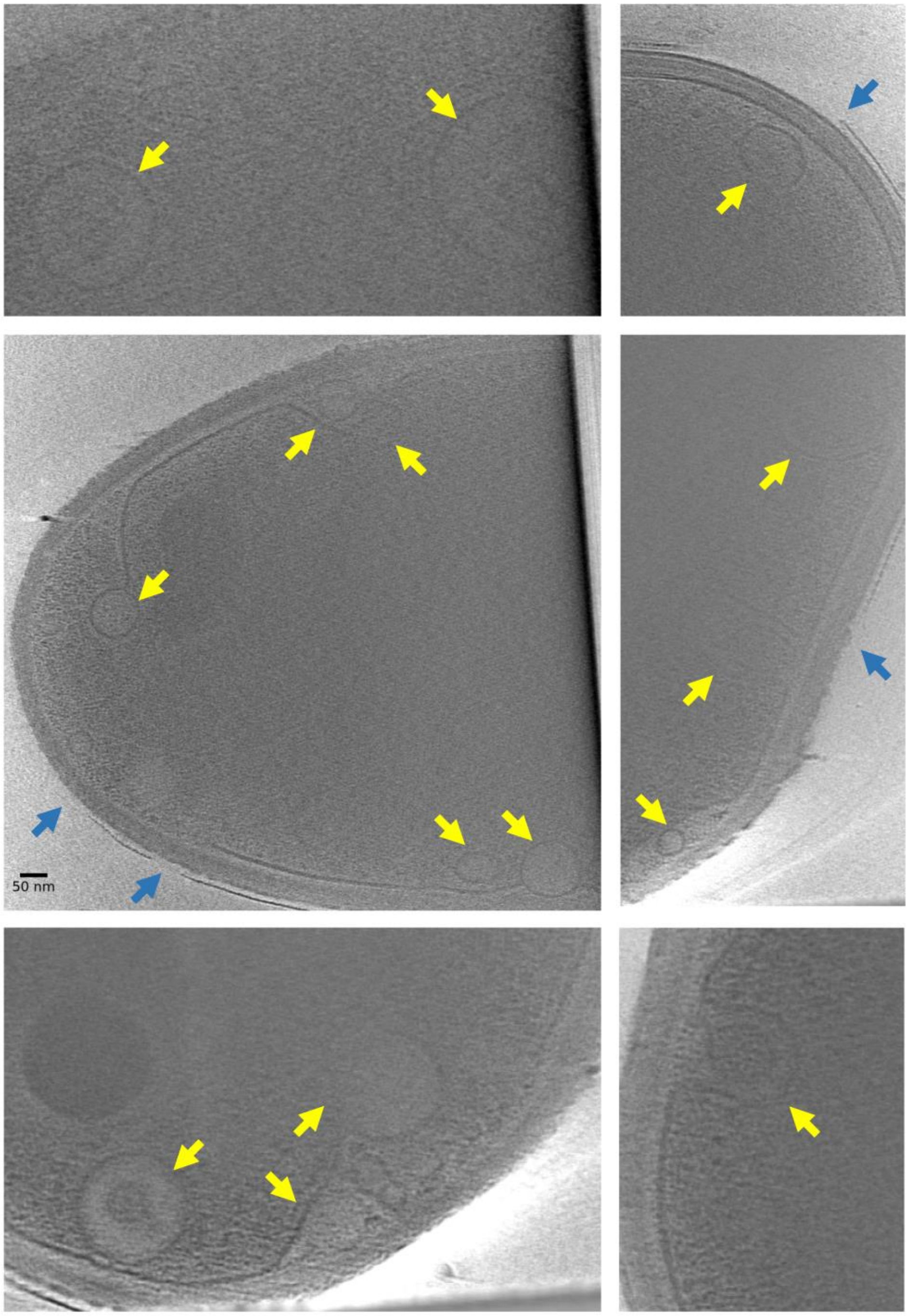

Fig. S6. Additional cryo-electron tomographic slices of *C. glutamicum* wild type cells treated with 4 x MIC A37 and an untreated control. Yellow arrows indicate locations of intracellular vesicle formation. Blue arrows indicate locations of mycolic acid layer detachment.

Table S1. Strains used in this work.

Strain	Information	Source or Reference
S. epidermidis A37	A37 producer strain	this work
S. epidermidis ATCC 14990	Type strain	[11]
C. glutamicum DSM 20300	Type strain	[12]
C. glutamicum A37/8	*C. glutamicum* DSM 20300 serial passaging mutant	this work
C. accolens 23	human nasal isolate	Laura Camus (LaCa) Collection /Tübingen - Infection biology
C. accolens 42	human nasal isolate	Laura Camus (LaCa) Collection /Tübingen - Infection biology
C. accolens 108	human nasal isolate	Laura Camus (LaCa) Collection /Tübingen - Infection biology
C. accolens 143	human nasal isolate	Laura Camus (LaCa) Collection /Tübingen - Infection biology
C. accolens 157	human nasal isolate	Laura Camus (LaCa) Collection /Tübingen - Infection biology
C. accolens 173	human nasal isolate	Laura Camus (LaCa) Collection /Tübingen - Infection biology
C. accolens 192	human nasal isolate	Laura Camus (LaCa) Collection /Tübingen - Infection biology
C. accolens 221	human nasal isolate	Laura Camus (LaCa) Collection /Tübingen - Infection biology
C. accolens 249	human nasal isolate	Laura Camus (LaCa) Collection /Tübingen - Infection biology
C. accolens 268	human nasal isolate	Laura Camus (LaCa) Collection /Tübingen - Infection biology

C. aurimucosum M5	human nasal isolate	[13]
C. aurimucosum M7	human nasal isolate	[13]
C. kefirresidentii 100	human nasal isolate	Laura Camus (LaCa) Collection /Tübingen - Infection biology
*C. kefirresidentii*223	human nasal isolate	Laura Camus (LaCa) Collection /Tübingen - Infection biology
C. kefirresidentii 54	human nasal isolate	Laura Camus (LaCa) Collection /Tübingen - Infection biology
C. kefirresidentii 62	human nasal isolate	Laura Camus (LaCa) Collection /Tübingen - Infection biology
C. propinquum 15	human nasal isolate	Laura Camus (LaCa) Collection /Tübingen - Infection biology
C. propinquum 70	human nasal isolate	Laura Camus (LaCa) Collection /Tübingen - Infection biology
C. propinquum 170	human nasal isolate	Laura Camus (LaCa) Collection /Tübingen - Infection biology
C. propinquum 263	human nasal isolate	Laura Camus (LaCa) Collection /Tübingen - Infection biology
C. propinquum 265	human nasal isolate	Laura Camus (LaCa) Collection /Tübingen - Infection biology
C. pseudodiphteriticum 242	human nasal isolate	Laura Camus (LaCa) Collection /Tübingen - Infection biology
C. pseudodiphteriticum 243	human nasal isolate	Laura Camus (LaCa) Collection /Tübingen - Infection biology
C. pseudodiphteriticum 244	human nasal isolate	Laura Camus (LaCa) Collection /Tübingen - Infection biology
C. simulans M18	human nasal isolate	[13]
C. simulans M22	human nasal isolate	[13]

C. simulans M43	human nasal isolate	[13]
C. simulans M60	human nasal isolate	[13]
C. kroppenstedtii M45	Human nasal isolate	[13]
C. tuberculostearicum 102	human nasal isolate	Laura Camus (LaCa) Collection /Tübingen - Infection biology
C. tuberculostearicum 117	human nasal isolate	Laura Camus (LaCa) Collection /Tübingen - Infection biology
C. tuberculostearicum 204	human nasal isolate	Laura Camus (LaCa) Collection /Tübingen - Infection biology
C. tuberculostearicum 205	human nasal isolate	Laura Camus (LaCa) Collection /Tübingen - Infection biology
C. tuberculostearicum 206	human nasal isolate	Laura Camus (LaCa) Collection /Tübingen - Infection biology
C. tuberculostearicum 224	human nasal isolate	Laura Camus (LaCa) Collection /Tübingen - Infection biology
C. tuberculostearicum 55	human nasal isolate	Laura Camus (LaCa) Collection /Tübingen - Infection biology
C. tuberculostearicum 63	human nasal isolate	Laura Camus (LaCa) Collection /Tübingen - Infection biology
C. tuberculostearicum 7	human nasal isolate	Laura Camus (LaCa) Collection /Tübingen - Infection biology
C. tuberculostearicum 9	human nasal isolate	Laura Camus (LaCa) Collection /Tübingen - Infection biology
Corynebacterium accolens 63VAs_B8	human nasal isolate	[13]
Corynebacterium aurimucosum 10VPs_Sm8	human nasal isolate	[13]
Corynebacterium kroppenstedtii 82VAs_B6	human nasal isolate	[13]

Corynebacterium propinquum 63VAs_B4	human nasal isolate	[13]
Corynebacterium pseudodiphteriticum 90VAs_B3	human nasal isolate	[13]
Corynebacterium simulans 81MNs_B1	human nasal isolate	[13]
Corynebacterium striatum 50MNs_Sm2	human nasal isolate	[13]
Mycobacterium bovis BCG	live vaccine strain	[14]
Staphylococcus aureus RN4220	laboratory strain	[15]
Staphylococcus aureus SA113	laboratory strain	[16]
Staphylococcus aureus COL	clinical isolate, MRSA	[17]
Staphylococcus aureus USA300 JE2	MRSA	[18]
Staphylococcus capitis 15-B10536	clinical isolate	[19]
Staphylococcus epidermidis ATCC12228		ATCC
Staphylococcus sciuri 1	clinical isolate	[19]
Stapylococcus simulans 22		[20]
Staphylococcus warneri 15-O10013	clinical isolate	[19]
Bacillus subtilis 168		[21]
Escherichia coli K12 W3110		[22]

Table S2. Overview of all publicly available epilancin assemblies.

ID	Strain	Species	Accession Number	Contig	Isolation source	Country	BioProject	Publication
15X	15X154	*S. epidermidis*	JQ979180.1	--	--	--	--	[23]
A37	A37	*S. epidermidis*	SAMN36827138	CP133000	nasal swab; *Homo sapiens*	DE	PRJNA801128	This work
K7	SCK7	*S. casei*	SAMN36827139	CP133008	nasal swab; *Homo sapiens*	--	PRJNA801128	This work
--	MI 10-1553	*S. ursi****	GCA_010365305.1	CP048279.1	oral lip fold; bear	US	PRJNA602989	--
EC1	APC 3775	*S. epidermidis*	GCA_009897805.1	NZ_SHGA01000021.1	human milk	IE	PRJNA521309	[24]
EC1	APC 3810	*S. epidermidis*	GCA_009897825.1	NZ_SHGB01000020.1	human milk	IE	PRJNA521309	[24]
EC2	DE0273	*S. epidermidis*	GCA_007678005.1	NZ_VEDU01000025.1	--	US	PRJNA543692	[25]
EC3	DE0273	*S. epidermidis*	GCA_007677005.1	NZ_VEBW01000023.1	--	US	PRJNA543693	[25]
EC3	DE0274	*S. epidermidis*	GCA_007676825.1	VEBO01000023.1	--	US	PRJNA543694	[25]
EC3 ****	HD43N3	*S. epidermidis*	GCA_016883745.1	JACMAV010000002	nose; *Homo sapiens*	DE	PRJNA546129	[26]
EC3	A5	*S. epidermidis*	GCA_004329135.1	NZ_SCHA01000018.1	skin; *Homo sapiens*	US	PRJNA514867	--

EC4	DE0276	*S. epidermidis*	GCA_007677025.1	NZ_VEBU01000023.1	--	US	PRJNA543694	[25]
EC5	BR2786_aL	*S. agnetis*	GCA_023925115.1	JALGOP010000032.1	skin; bovine	AU	PRJNA809943	--
EC6	NCTC7887-sc-2371519	*S. agnetis*	GCA_900457735.1	UHAH01000001.1	--	--	PRJEB6403	--
EC7	H69	*S. hominis*	GCA_001650655.1	LVVO01000111.1	air	US	PRJNA316465	--
EC7	p3-SID721	*S. hominis*	GCA_025143205.1	JALXNI010000013.1	skin; *Homo sapiens*	US	PRJNA803478	--
EC7*	ATCC 51192	*Shewanella algae*	GCA_012396675.1	JAAXPX010000028.1	red alga	FR	PRJNA622446	--
EC8	BBMGS-S01-101	*S. hominis*	GCA_017850155.1	JAFIJA010000028.1	skin; *Homo sapiens*	JP	PRJNA692334	[27]
EC9	1H9	*S. hominis*	GCA_018408065.1	JAGSWT010000019.1	middle ear infection; *Homo sapiens*	UK	PRJNA722783	--
EC10	P912	*S. warneri*	GCA_004570505.1	SPPM01000017.1	oral swab; mouse	UK	PRJNA527079	--
EC10**	19428wF1_P912	*S. warneri*	GCA_015235035.1	JADGMF010000017.1	oral swab; mouse	UK	PRJNA671681	--
EC11	Ani-LG-057	*S. warneri*	GCA_021366815.1	JAHCPQ010000010.1	bovine milk	BE	PRJNA609060	[28]
EC12	X2969	*S. warneri*	SAMN35174365	--	chicken	ES	PRJNA974190	[29]

EC13 *****	8SE	*S. pasteuri*	GCA_021728465.1	JAKKDY010000013.1	table surface	SD	PRJNA767482	--
EC14	47-83	*S. xylosus*	GCA_900098615.1	FMRS01000009.1	bull milk	UK	PRJEB2655	[30]

*precursor peptide matches the respective cluster
**duplicate of EC10
***unofficial classification from TYGS
****large multi-isolate projects excluded from cluster search
*****classified as *S. warneri* in NCBI

Table S3. Single nucleotide polymorphisms in *C. glutamicum* A37/8.

ContigRef	PosRef	Ref	Alt	AAref	AAalt	ProteinID (ref)	GeneLen	ContigAlt	PosAlt	LenRef	LenAlt	Direction	Gene Description
10	36421	C	T	P	L	BCDO-EBAP _02171	2151	11	102130	138550	138550	-1	acyltransferase family protein
36	858	T	C	L	L	ORF1: comp (+3 – 1148+)	1146	30	293	1150	1150	-1	HNH endonuclease, partial
				Stop	Q	ORF2: 861-1148+	288						hyp. protein
36	960	G	T	G	C	ORF2: 861-1148+	288	30	191	1150	1150	-1	hyp. protein
40	130	G	T	H	Q	ORF1: comp (82-423+)	342	34	130	424	424	1	IS6 family transposase
40	343	C	A	E	D	ORF1: comp (82-423+)	342	34	343	424	424	1	IS6 family transposase
				L	I	ORF2: 172-423+	252						hyp. protein

Table S4. MICs of several cationic antimicrobial substances against *C. glutamicum* DSM 20300 (WT) and A37/8.

	MIC (µg/mL)	
	***C. glutamicum* WT**	***C. glutamicum* A37/8**
**A37**	1	8
**Pep5**	16	32
**Mersacidin**	1	1
**Nisin**	4	8
**Daptomycin**	0.25	0.125
**Kanamycin**	0.125	0.125

Supplementary References

1. Ewels PA, Peltzer A, Fillinger S, Patel H, Alneberg J, Wilm A, *et al.* The nf-core framework for community-curated bioinformatics pipelines. *Nat Biotechnol* 2020; **38**: 276–278.
2. Li W, O'Neill KR, Haft DH, DiCuccio M, Chetvernin V, Badretdin A, *et al.* RefSeq: expanding the prokaryotic genome annotation pipeline reach with protein family model curation. *Nucleic Acids Res* 2021; **49**: D1020–D1028.
3. Kuznetsov A, Bollin CJ. NCBI genome workbench: desktop software for comparative genomics, visualization, and genbank data submission. In: Kazutaka K (ed.), Multiple Sequence Alignment Methods and Protocols. New York: Humana New York, 2021, 261–295.
4. Hagen WJH, Wan W, Briggs JAG. Implementation of a cryo-electron tomography tilt-scheme optimized for high resolution subtomogram averaging. *J Struct Biol* 2017; **197**: 191–198.
5. Agulleiro J-I, Fernandez J-J. Tomo3D 2.0 – Exploitation of Advanced Vector eXtensions (AVX) for 3D reconstruction. *J Struct Biol* 2015; **189**: 147–152.
6. Kremer JR, Mastronarde DN, McIntosh JR. Computer visualization of three-dimensional image data using IMOD. *J Struct Biol* 1996; **116**: 71–76.
7. Schneider CA, Rasband WS, Eliceiri KW. NIH Image to ImageJ: 25 years of image analysis. *Nat Methods 2012 9:7* 2012; **9**: 671–675.
8. Ducret A, Quardokus EM, Brun Y V. MicrobeJ, a tool for high throughput bacterial cell detection and quantitative analysis. *Nat Microbiol* 2016; **1**: 16077
9. Lord SJ, Velle KB, Dyche Mullins R, Fritz-Laylin LK. SuperPlots: Communicating reproducibility and variability in cell biology. *J Cell Biol* 2020: **219**: e202001064
10. Motulsky HJ, Brown RE. Detecting outliers when fitting data with nonlinear regression - A new method based on robust nonlinear regression and the false discovery rate. *BMC Bioinformatics* 2006; **7**: 1–20.
11. Hugh R, Ellis MA. The neotype strain for *Staphylococcus epidermidis* (Winslow and Winslow 1908) Evans 1916. *Int J Syst Bacteriol* 1968; **18**: 231–239.
12. Abe S, Takayama K-I, Kinoshita S. Taxonomical studies on glutamic acid-producing bacteria. *J Gen Appl Microbiol* 1967; **13**: 279–301.
13. Kaspar U, Kriegeskorte A, Schubert T, Peters G, Rudack C, Pieper DH, *et al.* The culturome of the human nose habitats reveals individual bacterial fingerprint patterns. *Environ Microbiol* 2016; **18**: 2130–2142.
14. Brosch R, Gordon S V., Buchrieser C, Pym AS, Garnier T, Cole ST. Comparative genomics uncovers large tandem chromosomal duplications in *Mycobacterium bovis* BCG pasteur. *Yeast* 2000; **1**: 111–123.
15. Kreiswirth BN, Löfdahl S, Betley MJ, O'Reilly M, Schlievert PM, Bergdoll MS, *et al.* The toxic shock syndrome exotoxin structural gene is not detectably transmitted by a prophage. *Nature* 1983; **305**: 709–712.
16. Novick RP. Genetic systems in Staphylococci. In: Miller JH (ed.), Methods in Enzymology. Amsterdam: Elsevier, 1991, 587–636.
17. de Jonge BL, Chang YS, Gage D, Tomasz A. Peptidoglycan composition of a highly methicillin-resistant *Staphylococcus aureus* strain. The role of penicillin binding protein 2A. *J Biol Chem* 1992; **267**: 11248–11254.
18. Diep BA, Gill SR, Chang RF, Phan TH, Chen JH, Davidson MG, *et al.* Complete genome sequence of USA300, an epidemic clone of community-acquired meticillin-resistant *Staphylococcus aureus*. *Lancet* 2006; **367**: 731–739.
19. Schuster D, Josten M, Janssen K, Bodenstein I, Albert C, Schallenberg A, *et al.* Detection of methicillin-resistant coagulase-negative staphylococci harboring the class A mec complex by MALDI-TOF mass spectrometry. *Int J Med Microbiol* 2018; **308**: 522–526.
20. Bierbaum G, Sahl HG. Autolytic system of *Staphylococcus simulans* 22: influence of cationic peptides on activity of N-acetylmuramoyl-L-alanine amidase. *J Bacteriol* 1987; **169**: 5452–5458.
21. Anagnostopoulos C, Spizizen J. Requirements for transformation in *Bacillus subtilis*. *J Bacteriol* 1961; **81**: 741–746.
22. Bachmann BJ. Pedigrees of Some Mutant Strains of *Escherichia coli* K-12. *Bacteriol rev* 1972; **36**: 525-557.
23. Velásquez JE, Zhang X, Van Der Donk WA. Biosynthesis of the antimicrobial peptide epilancin 15X and its N-terminal lactate. *Chem Biol* 2011; **18**: 857–867.

24. Angelopoulou A, Warda AK, O'Connor PM, Stockdale SR, Shkoporov AN, Field D, *et al.* Diverse bacteriocins produced by strains from the human milk microbiota. *Front Microbiol* 2020; **11**: 788
25. Zhang C, Song W, Ma HR, Peng X, Anderson DJ, Fowler VG, *et al.* Temporal encoding of bacterial identity and traits in growth dynamics. *Proc Natl Acad Sci USA.* 2020; **117**: 20202–20210.
26. Both A, Huang J, Qi M, Lausmann C, Weißelberg S, Büttner H, *et al.* Distinct clonal lineages and within-host diversification shape invasive *Staphylococcus epidermidis* populations. *PLoS Pathog* 2021; **17**: e1009304.
27. Arikawa K, Ide K, Kogawa M, Saeki T, Yoda T, Endoh T, *et al.* Recovery of strain-resolved genomes from human microbiome through an integration framework of single-cell genomics and metagenomics. *Microbiome* 2021; **9**: 202.
28. Fergestad ME, De Visscher A, L'Abee-Lund T, Tchamba CN, Mainil JG, Thiry D, *et al.* Antimicrobial resistance and virulence characteristics in 3 collections of staphylococci from bovine milk samples. *J Dairy Sci* 2021; **104**: 10250–10267.
29. Fernández-Fernández R, Elsherbini AMA, Lozano C, Martínez A, de Toro M, Zarazaga M, *et al.* Genomic analysis of bacteriocin-producing staphylococci: high prevalence of lanthipeptides and the micrococcin P1 biosynthetic gene clusters. *Probiotics Antimicrob Proteins* 2023.
30. Harrison EM, Paterson GK, Holden MTG, Larsen J, Stegger M, Larsen AR, *et al.* Whole genome sequencing identifies zoonotic transmission of MRSA isolates with the novel *mecA* homologue *mecC*. *EMBO Mol Med* 2013; **5**: 509–515.
